# Supplementary figures and images for: The GET pathway serves to activate Atg32-mediated mitophagy by ER targeting of the Ppg1-Far complex
Source: Life Sci Alliance. 2023 Jan 25;6(4):e202201640. doi: 10.26508/lsa.202201640 (PMC9880027; doi:10.26508/lsa.202201640)

Figure 1A

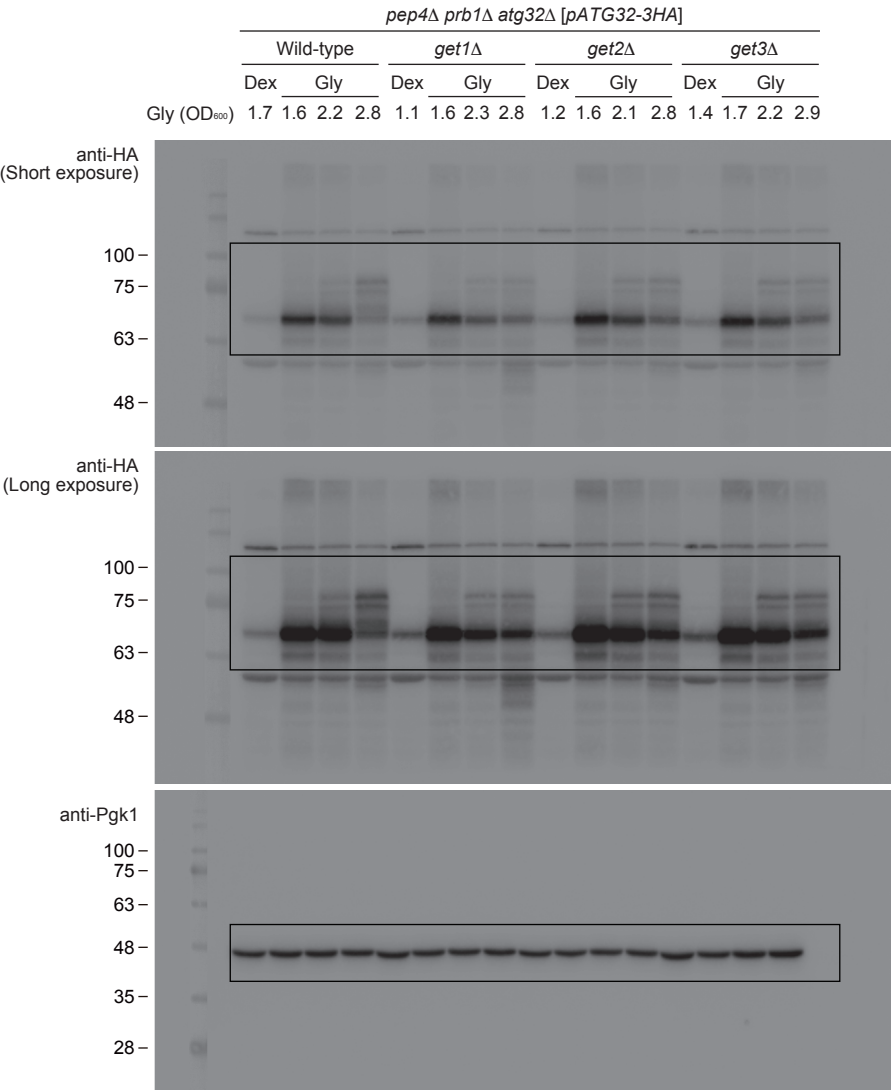

Figure 1C

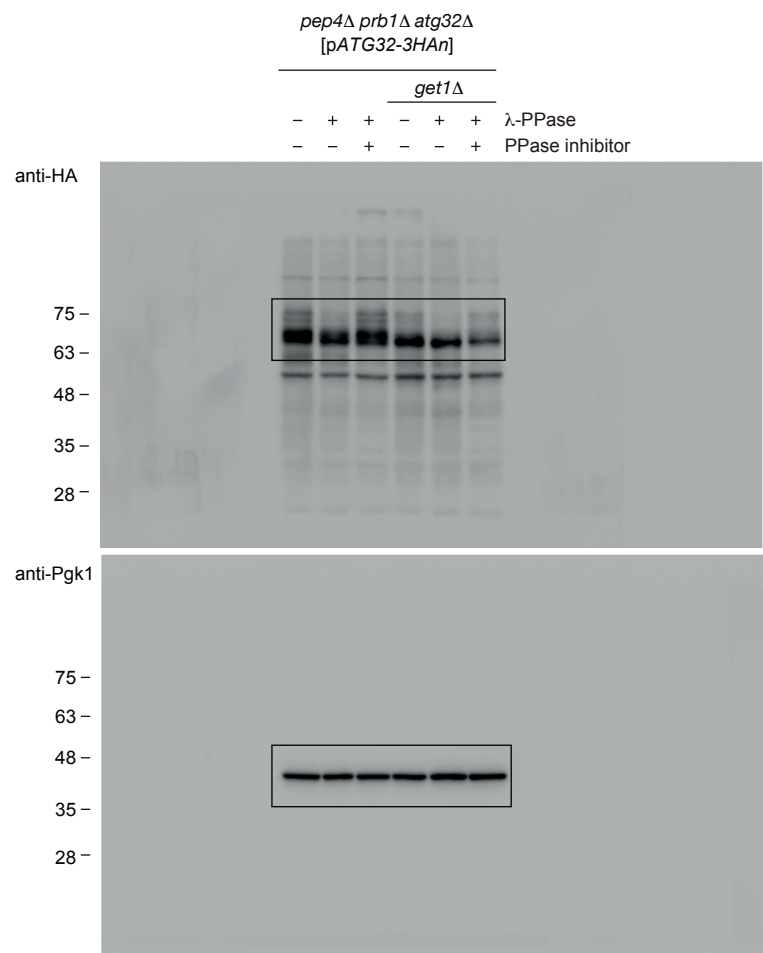

Supplement: Supplementary file 1 [file LSA-2022-01640_SdataF1.1.pdf]

Figure S1B

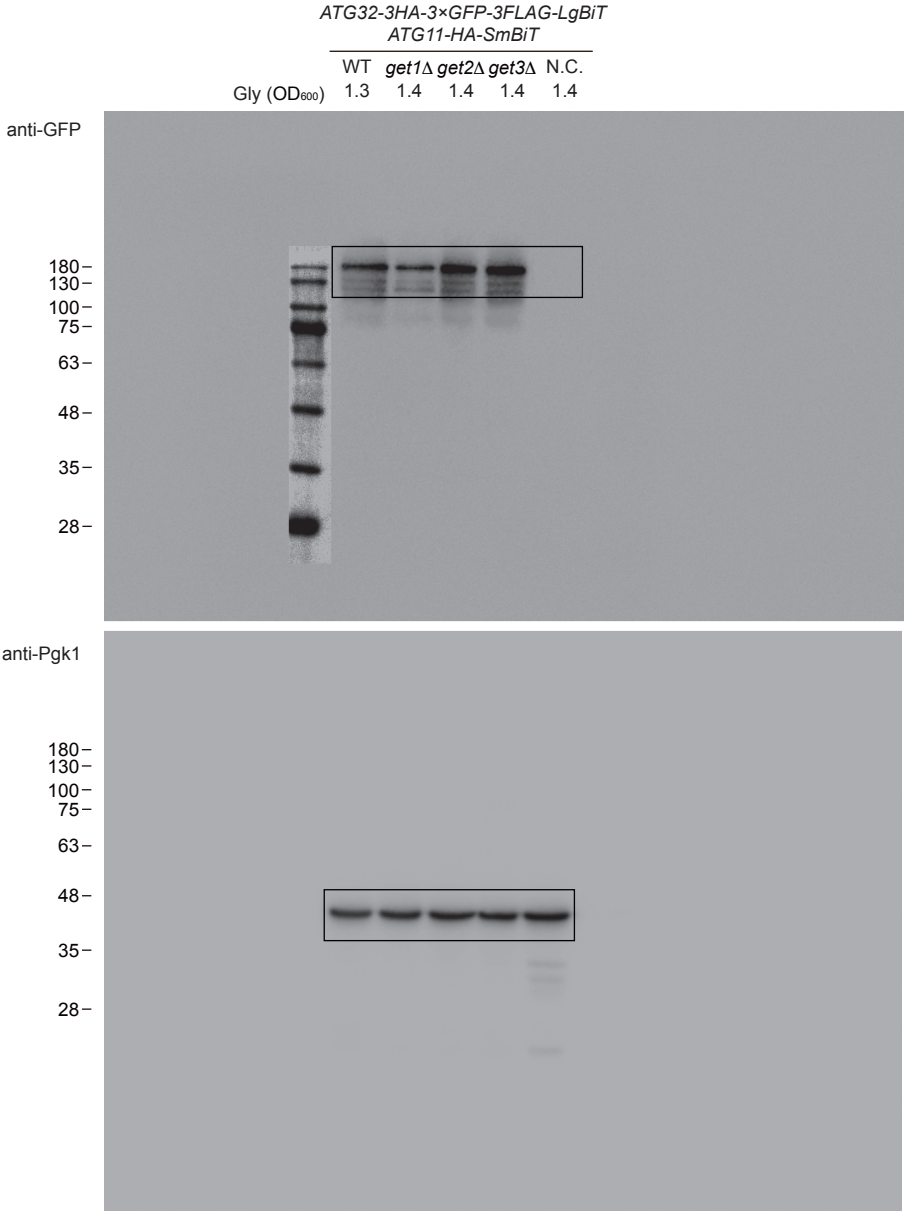

Figure S1C

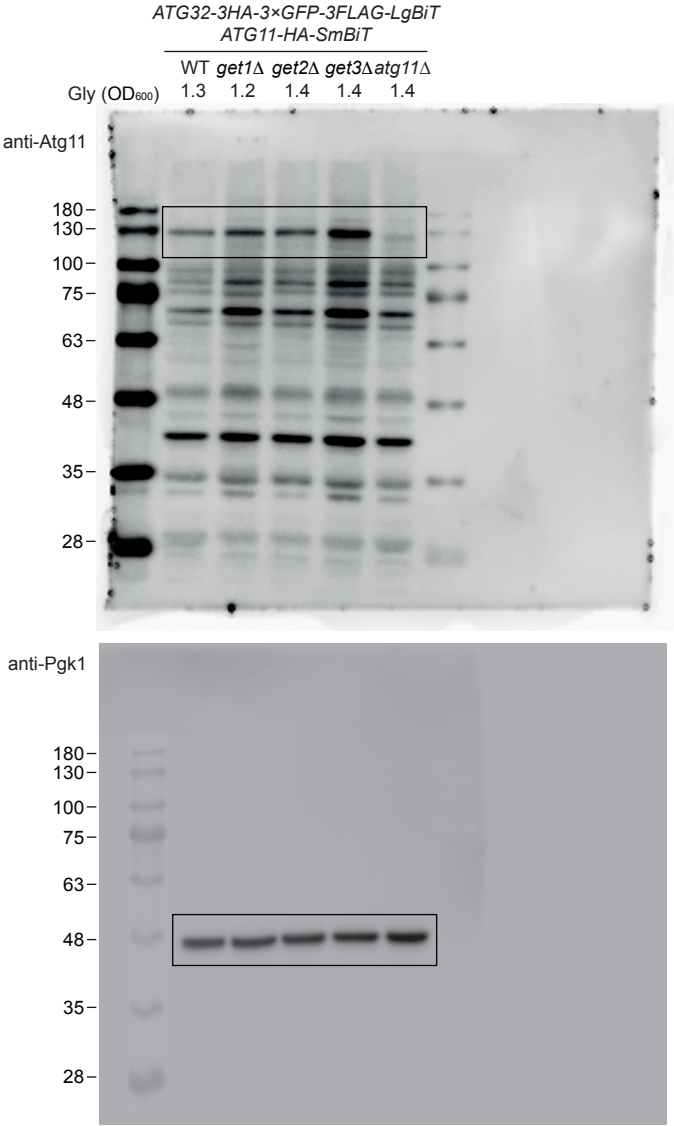

Figure S1E

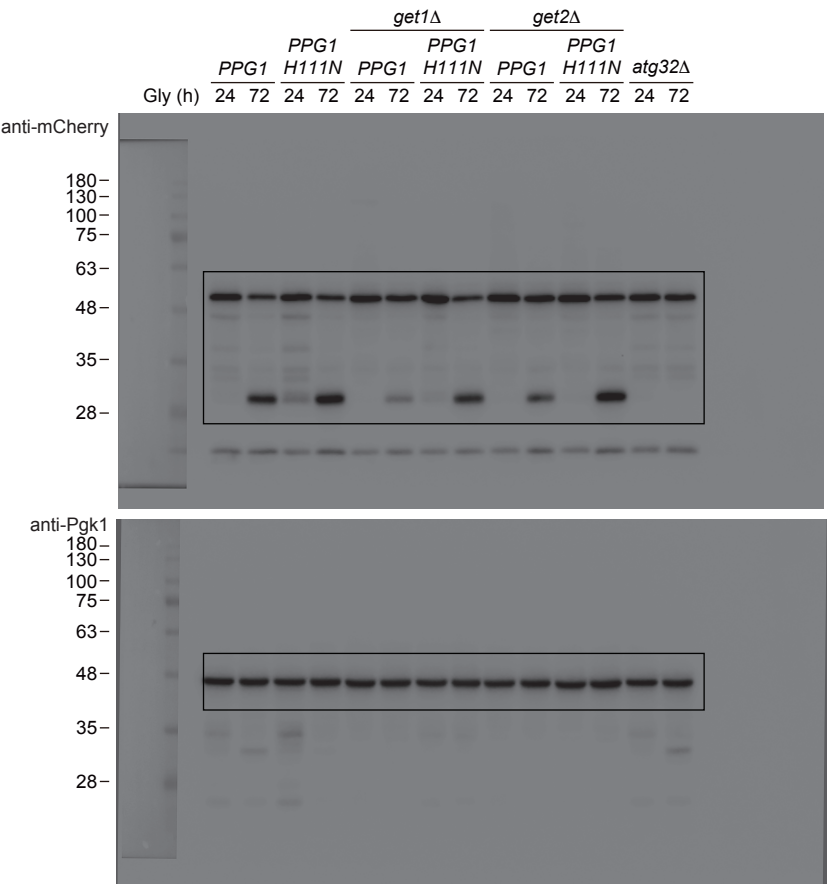

Figure S1G

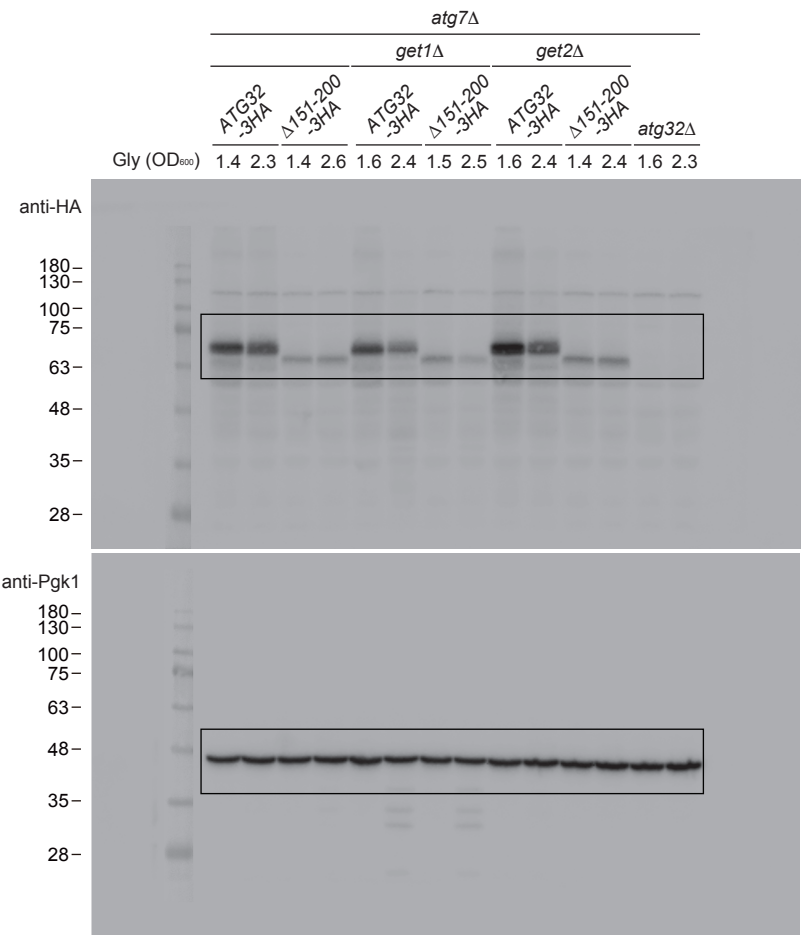

Supplement: Supplementary file 3 [file LSA-2022-01640_SdataFS1.1.pdf]

Figure 2B

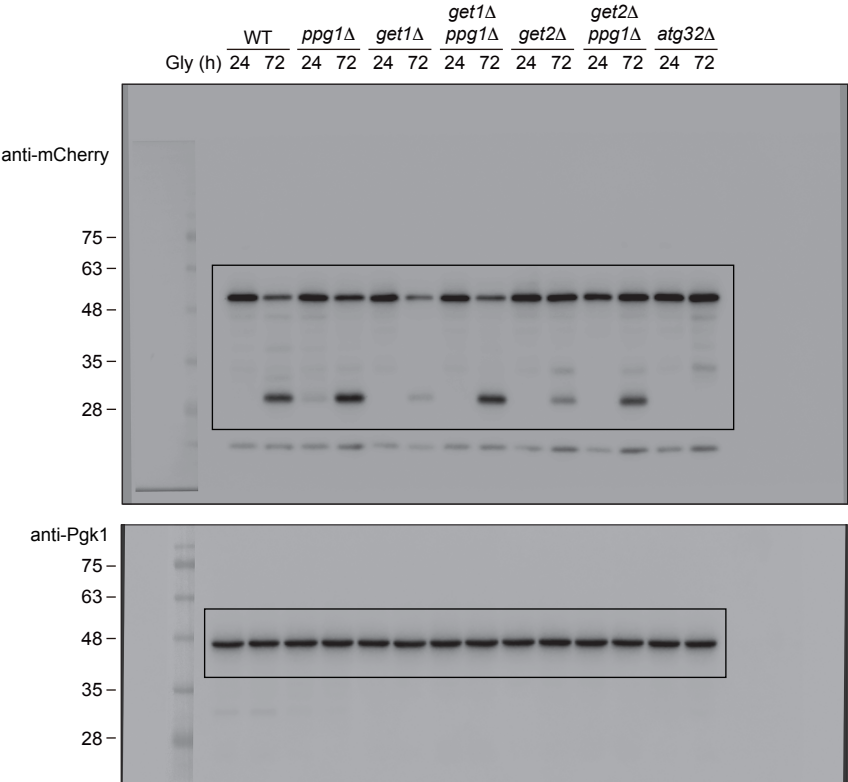

Figure 2E

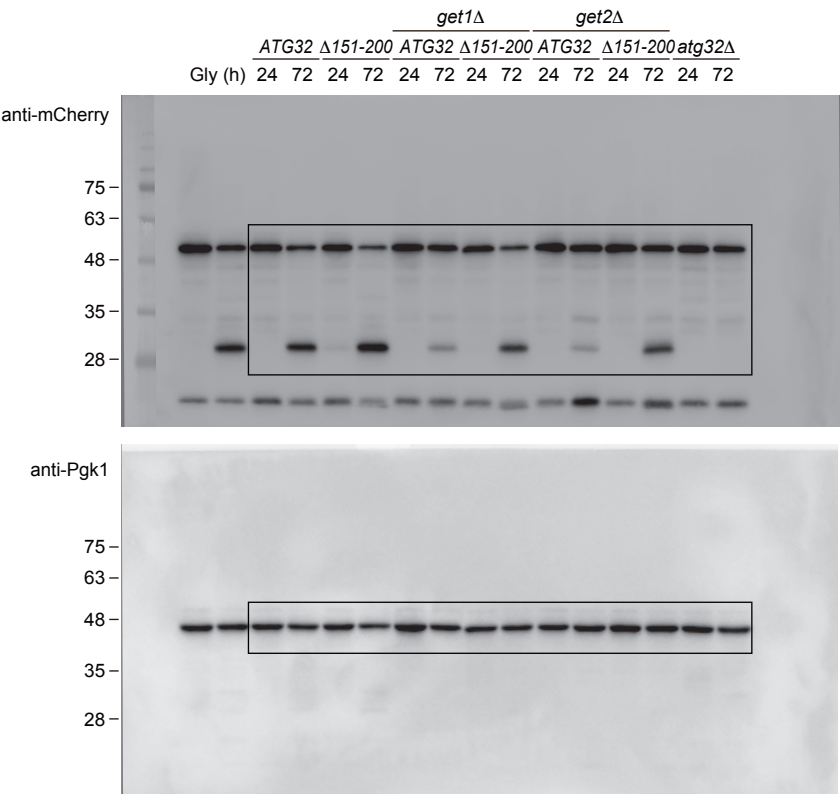

Supplement: Supplementary file 6 [file LSA-2022-01640_SdataF2.2.pdf]

Figure 4B

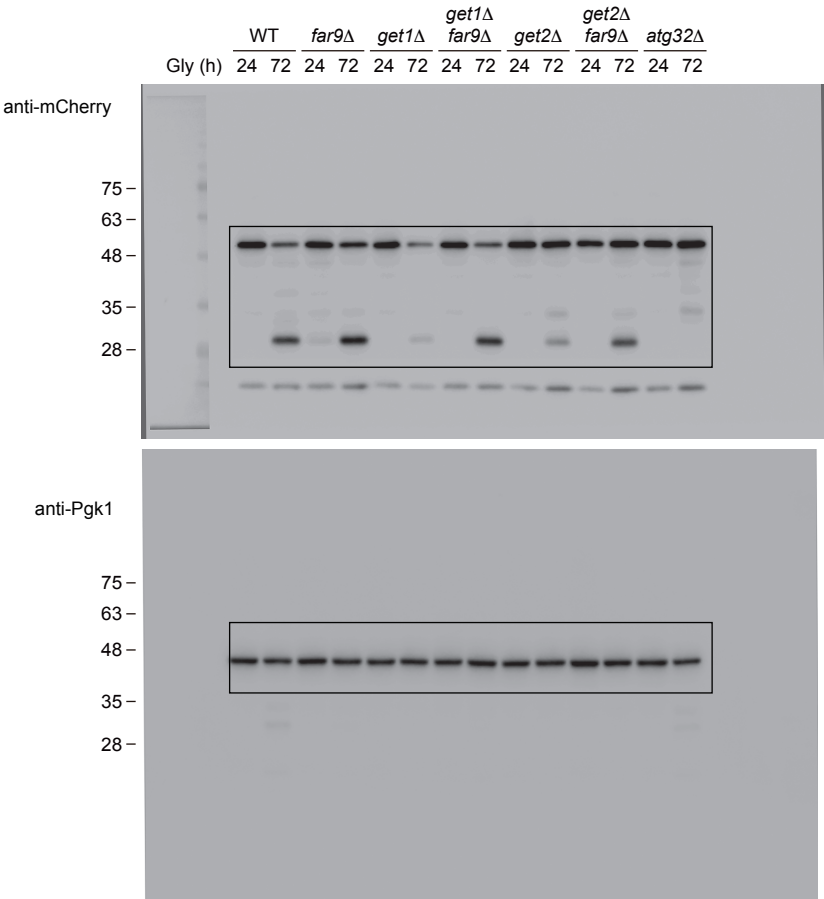

Supplement: Supplementary file 14 [file LSA-2022-01640_SdataF4.2.pdf]

Figure S4B

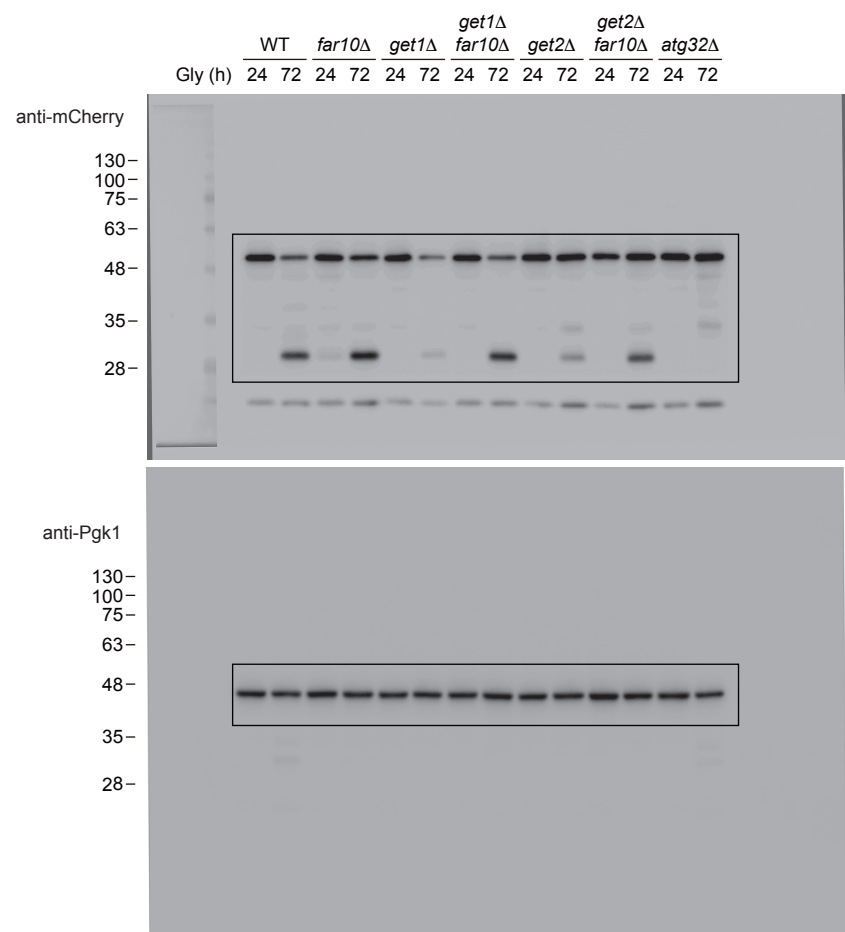

Supplement: Supplementary file 16 [file LSA-2022-01640_SdataFS4.2.pdf]

Figure 6A

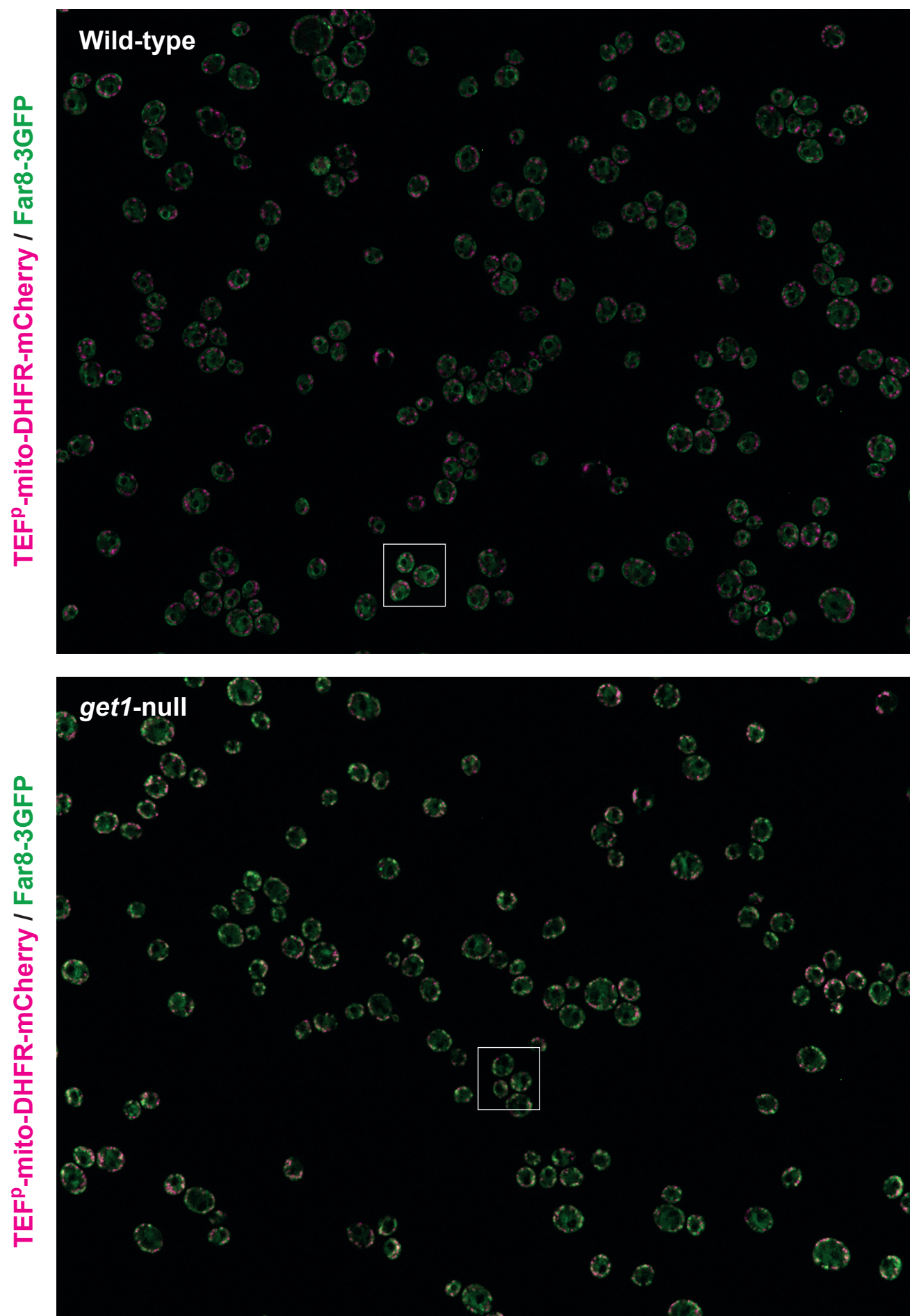

Figure 6A

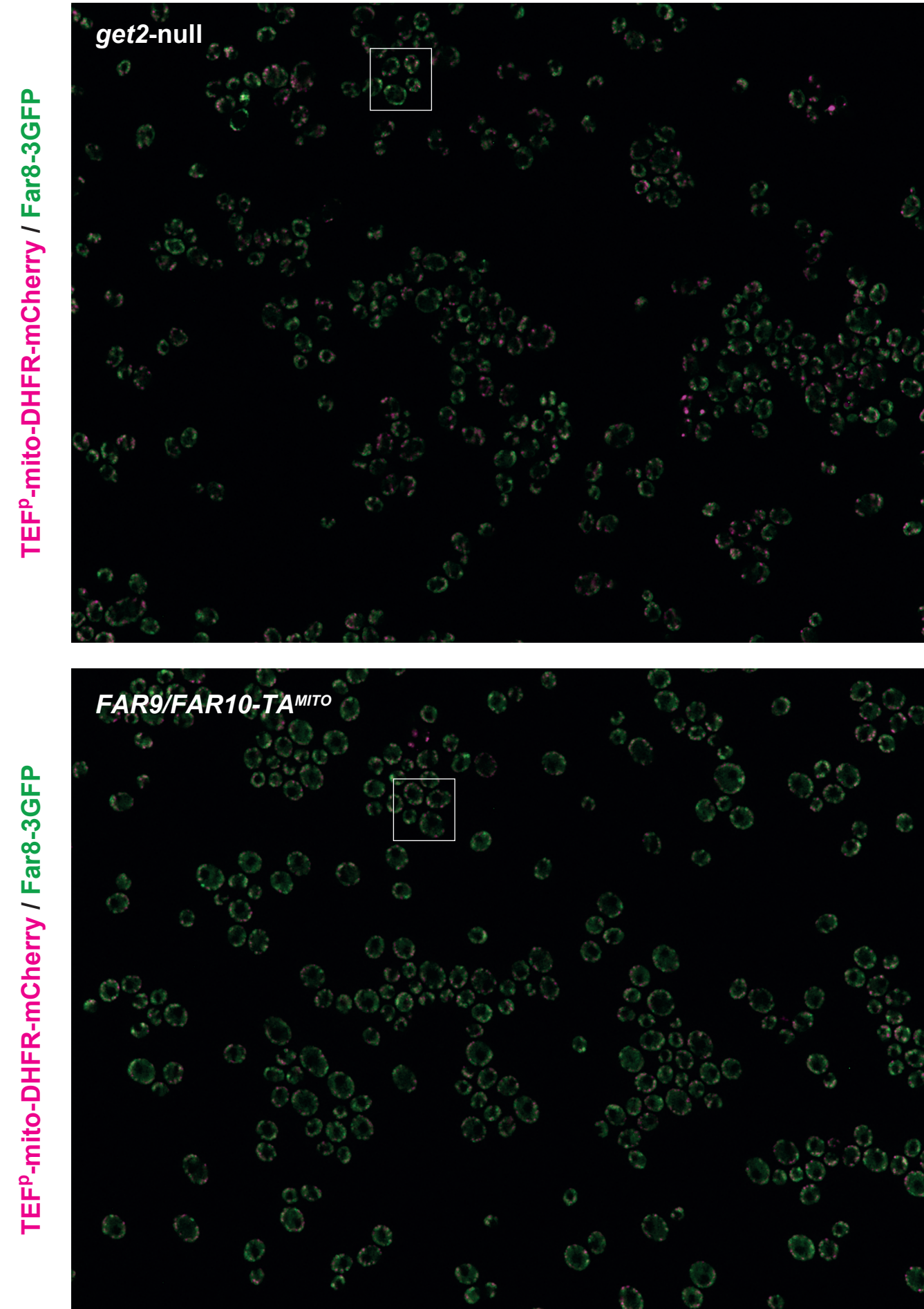

Figure 6D

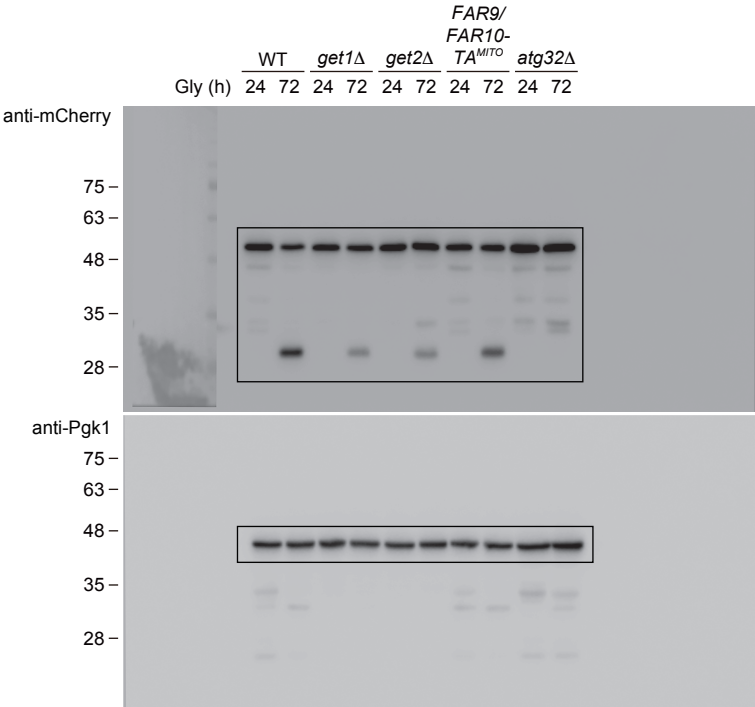

Figure 6F

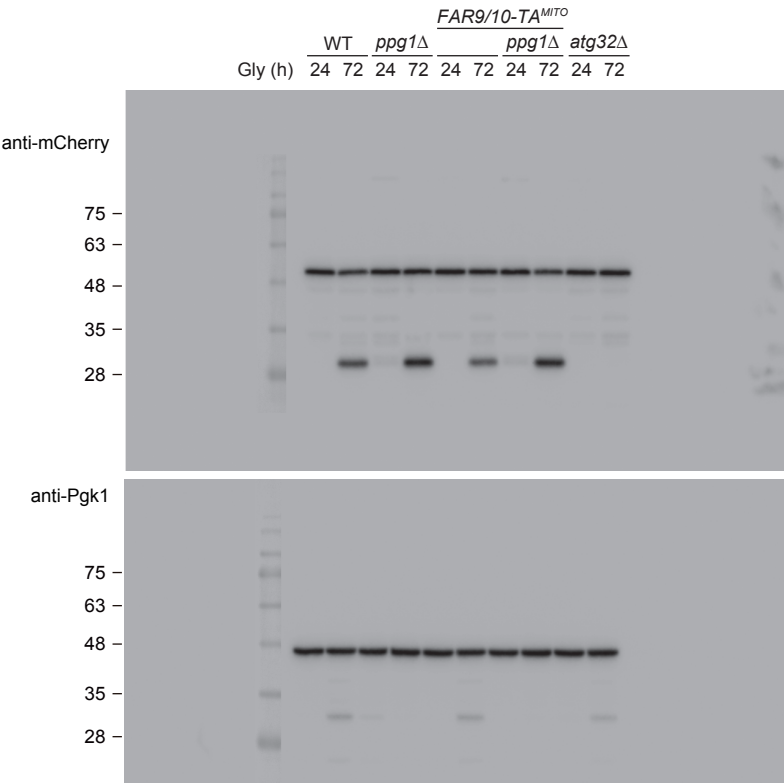

Supplement: Supplementary file 21 [file LSA-2022-01640_SdataF6.1.pdf]

Figure S6A

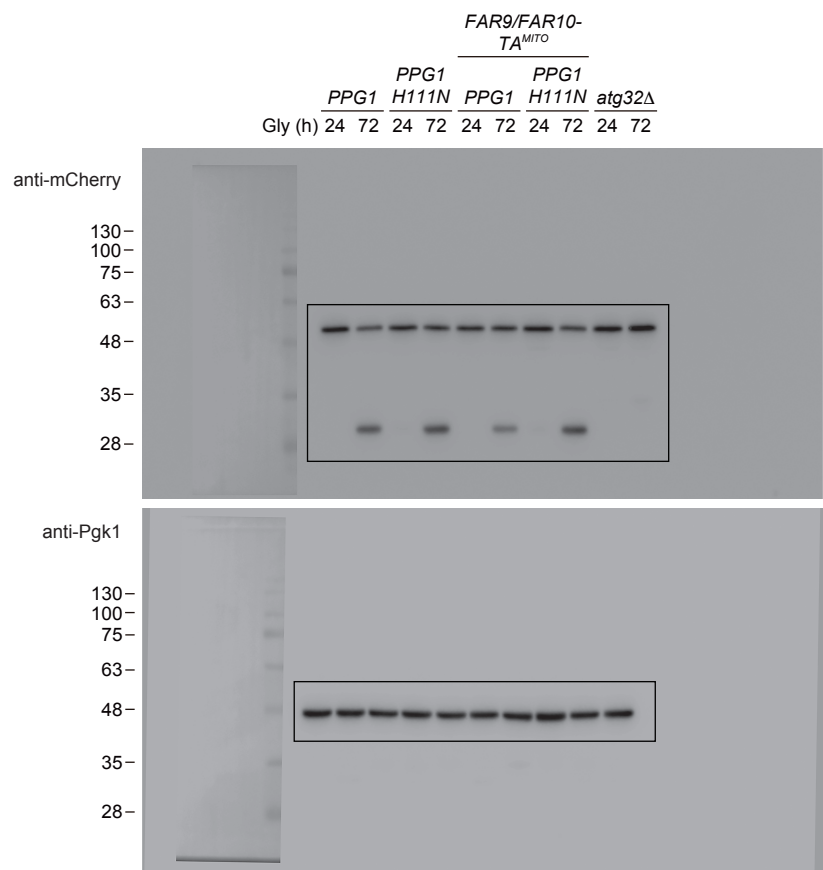

Figure S6D

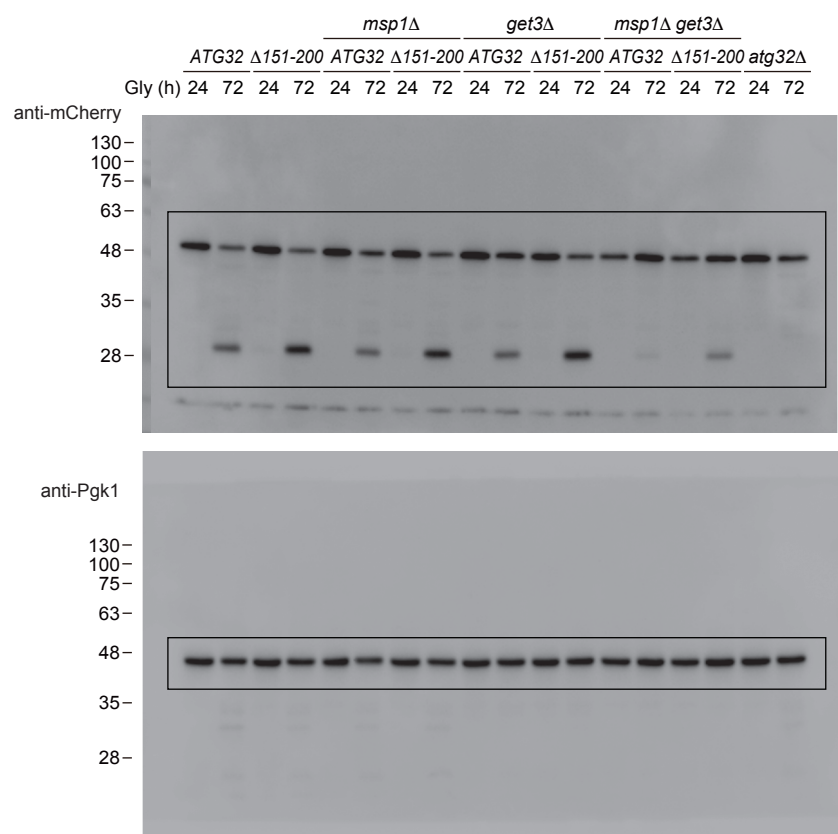

Supplement: Supplementary file 23 [file LSA-2022-01640_SdataFS6.1.pdf]
