# Supplementary material for: The GET pathway serves to activate Atg32-mediated mitophagy by ER targeting of the Ppg1-Far complex
Source: Life Sci Alliance. 2023 Jan 25;6(4):e202201640. doi: 10.26508/lsa.202201640 (PMC9880027; doi:10.26508/lsa.202201640)
Supplement: Supplementary file 7 [file LSA-2022-01640_SdataF3.1.pdf]

Onishi et al. Source data related to Figure 3

Figure 3A

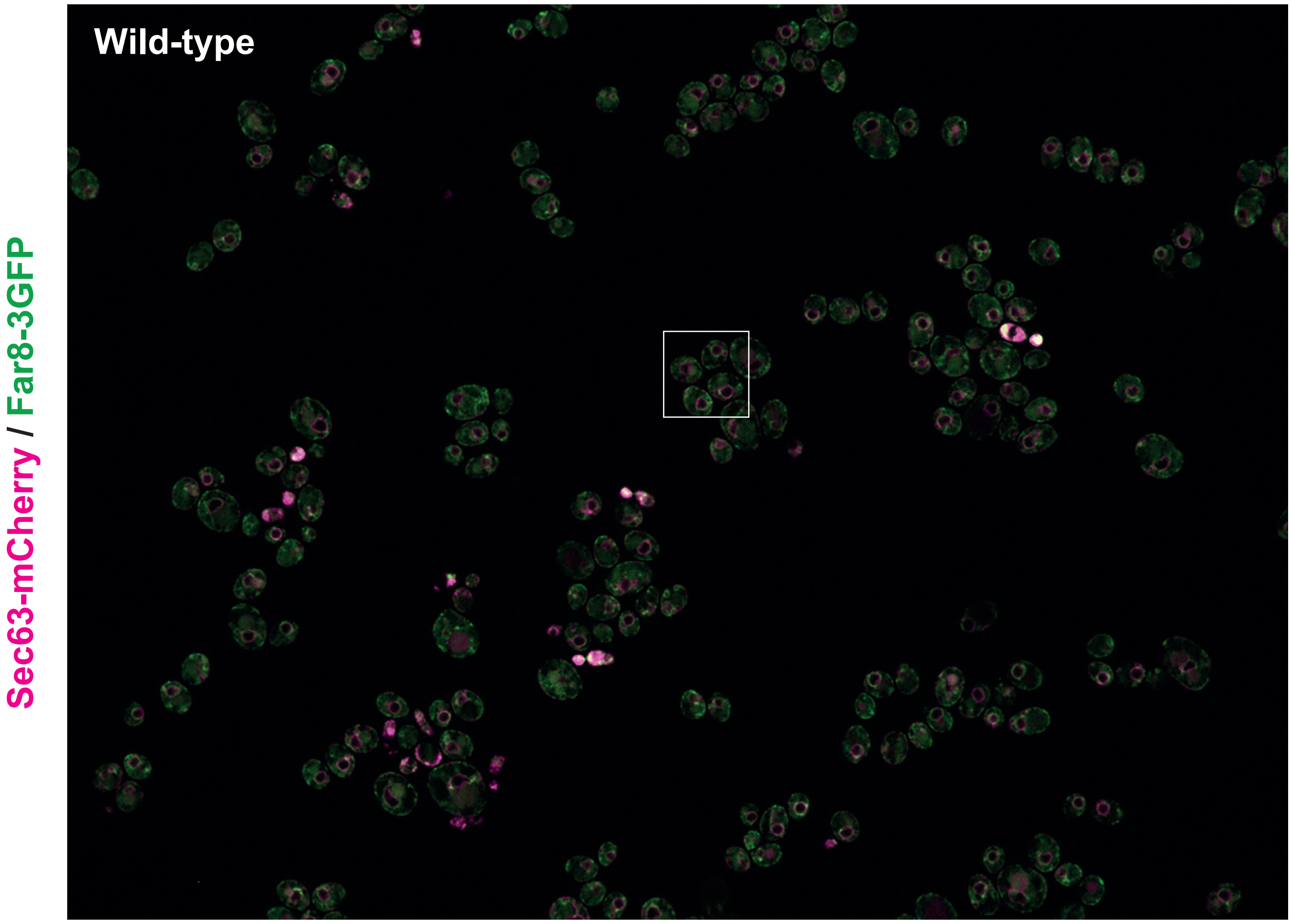

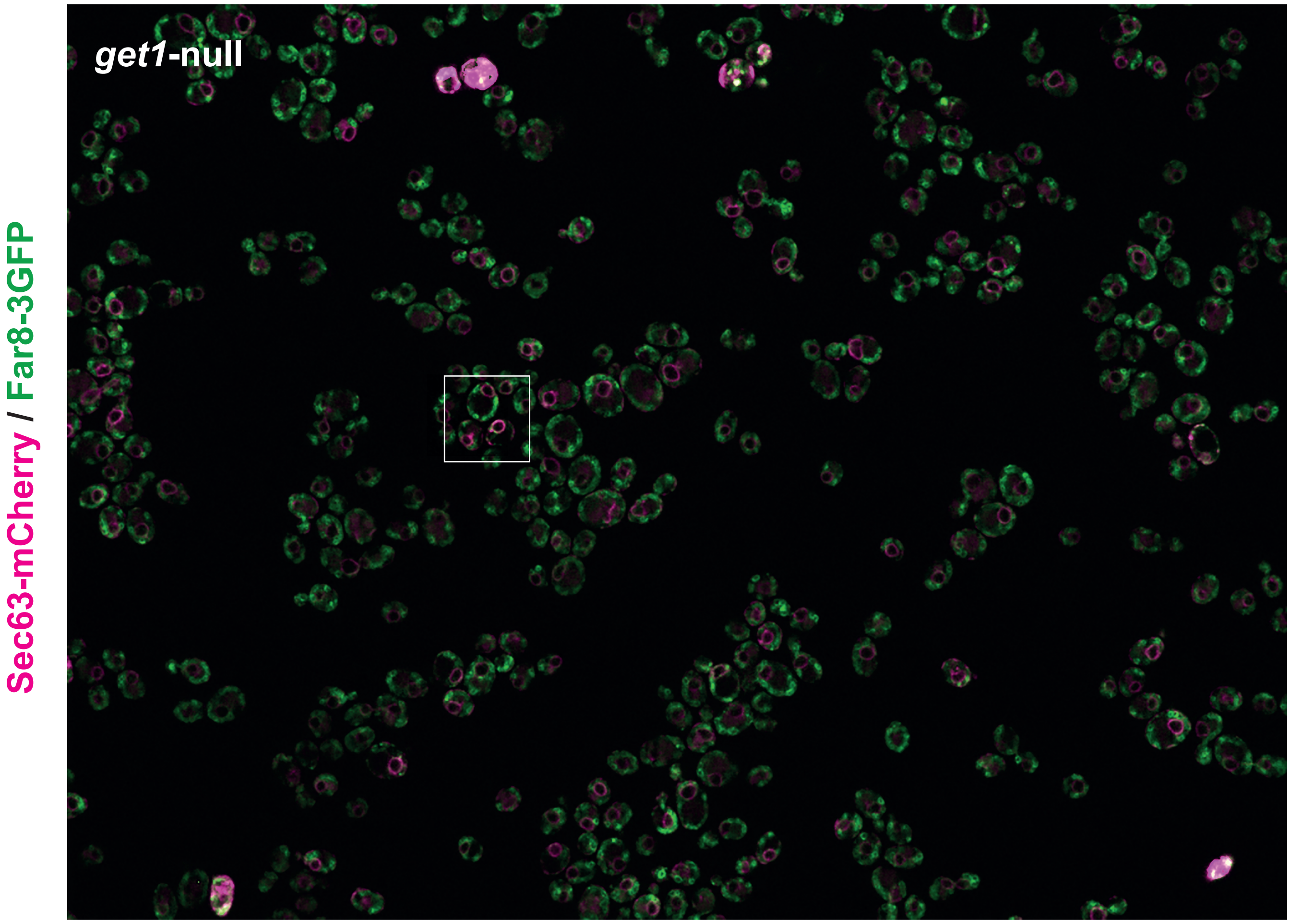

Figure 3C

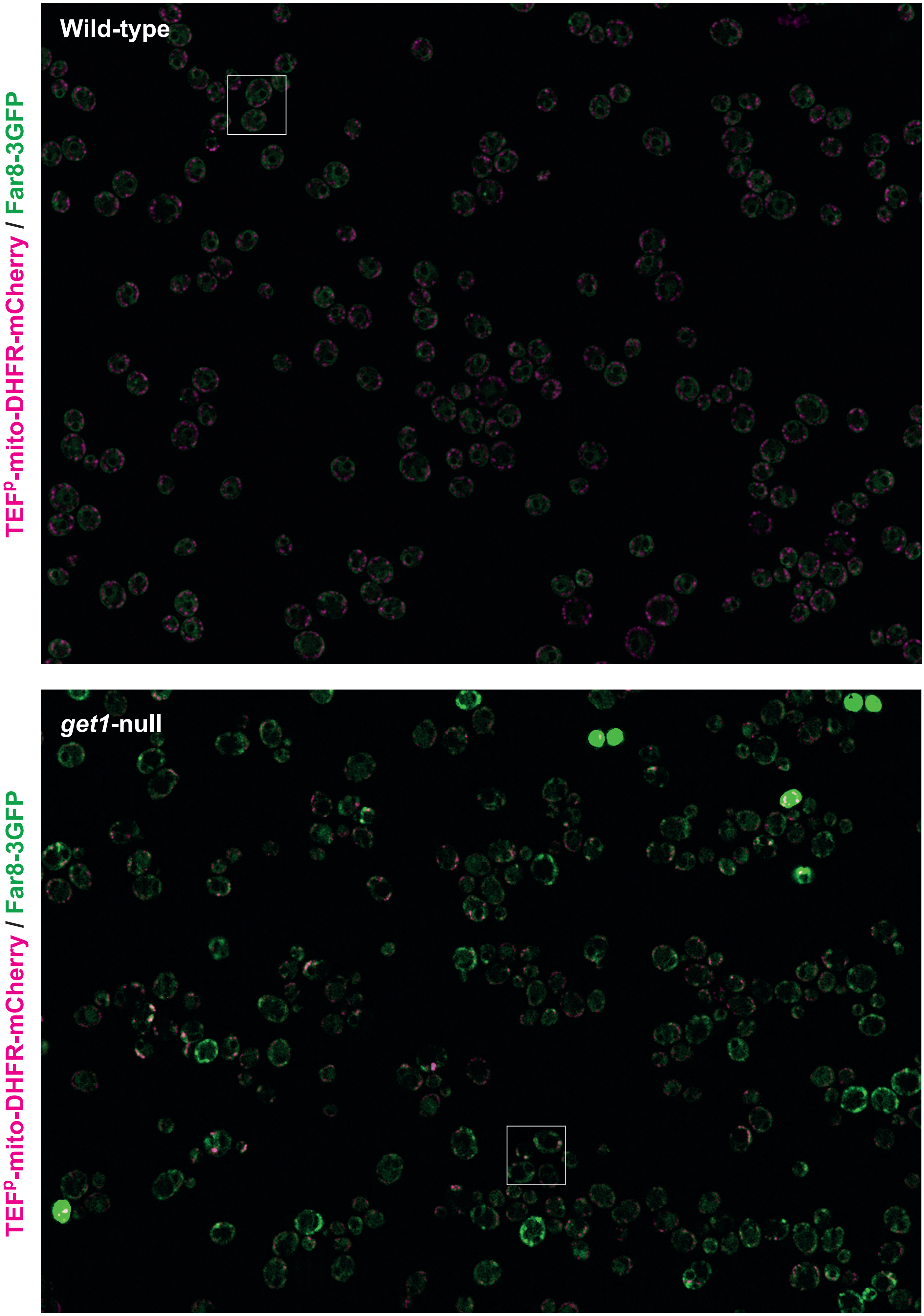
Wild-type
TEF^p-mito-DHFR-mCherry / Far8-3GFP
get1-null
TEF^p-mito-DHFR-mCherry / Far8-3GFP
