# Supplementary material for: The GET pathway serves to activate Atg32-mediated mitophagy by ER targeting of the Ppg1-Far complex
Source: Life Sci Alliance. 2023 Jan 25;6(4):e202201640. doi: 10.26508/lsa.202201640 (PMC9880027; doi:10.26508/lsa.202201640)
Supplement: Supplementary file 9 [file LSA-2022-01640_SdataFS2.1.pdf]

Onishi et al. Source data related to Figure S2

Figure S2A

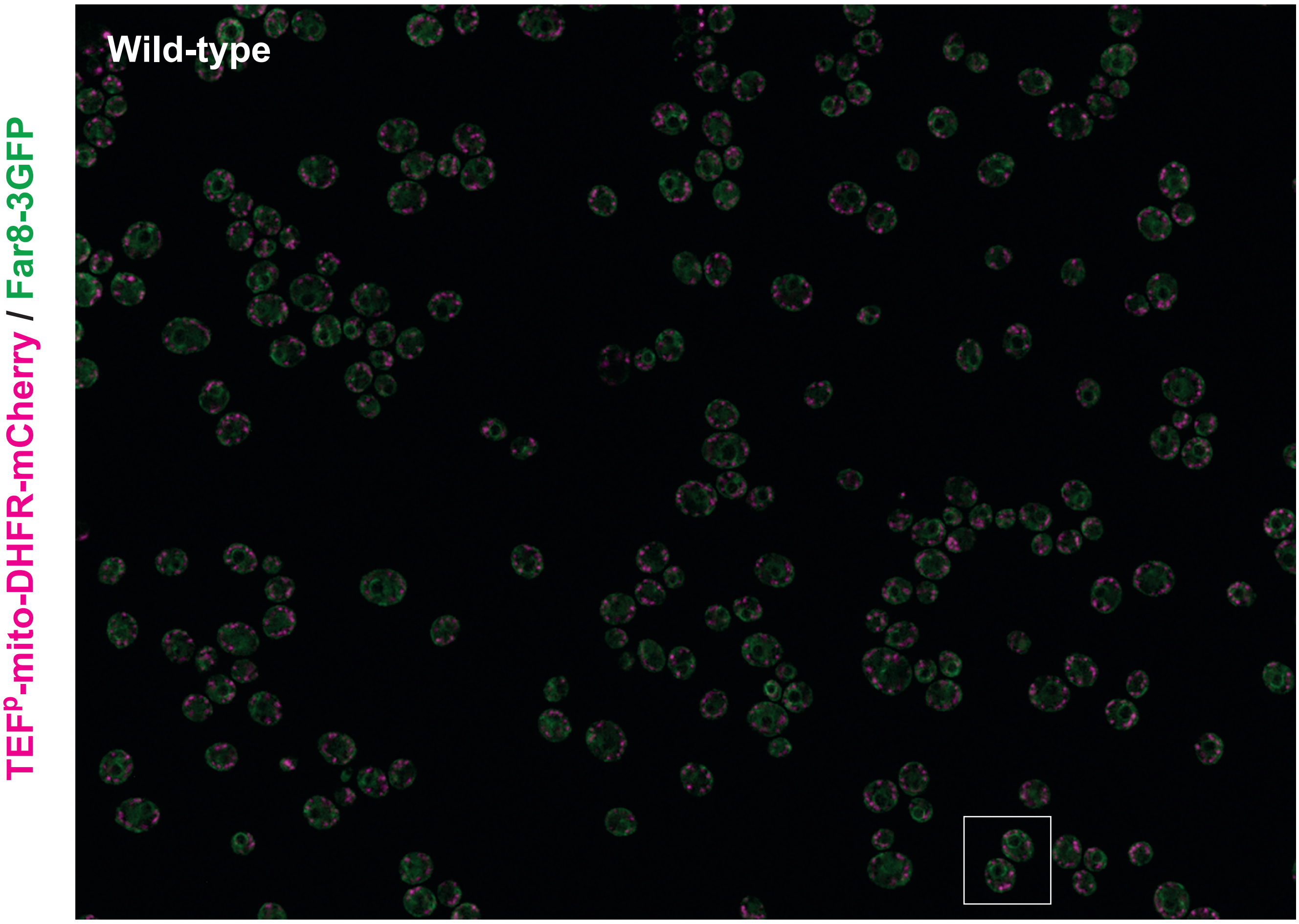

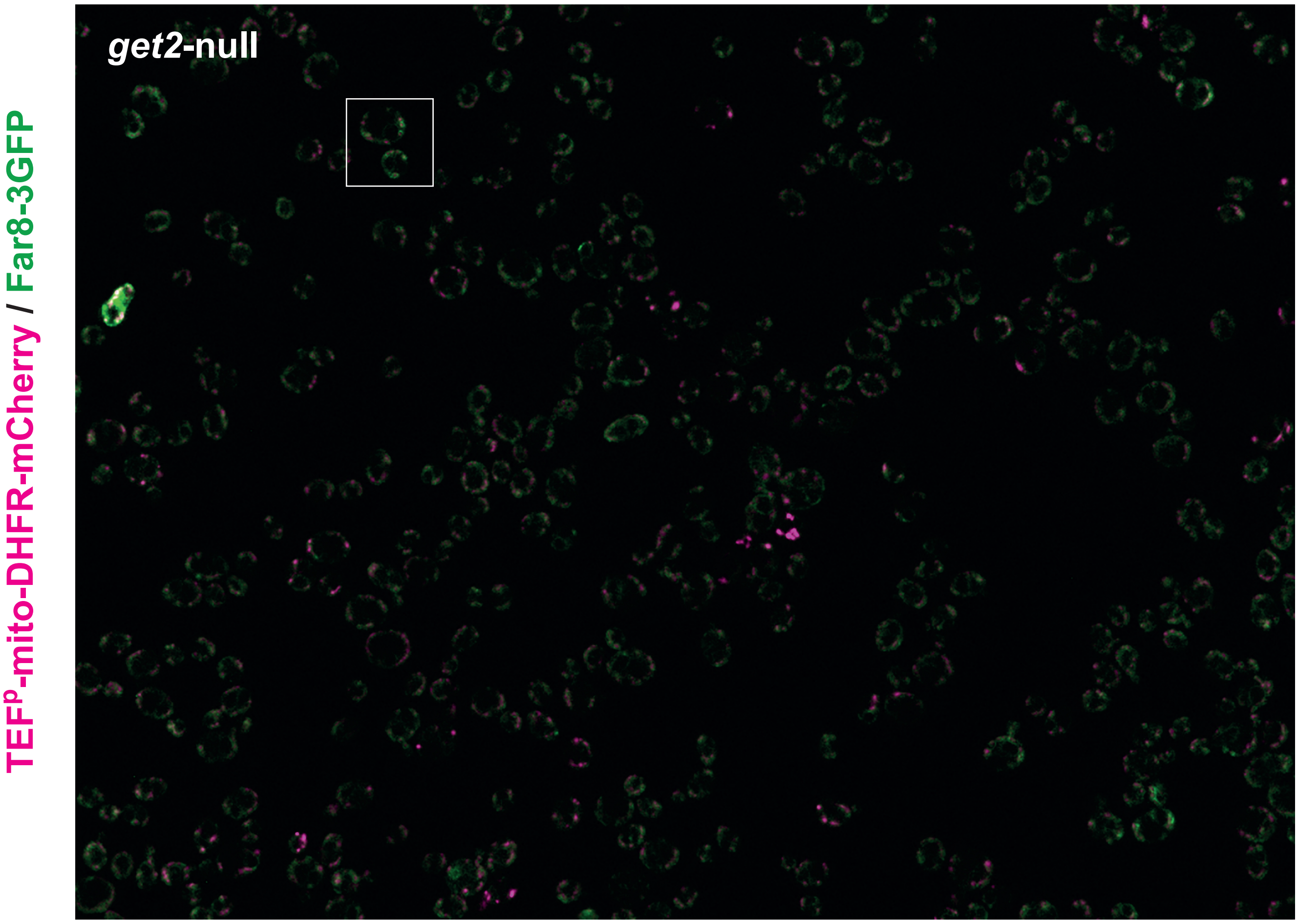

Figure S2A

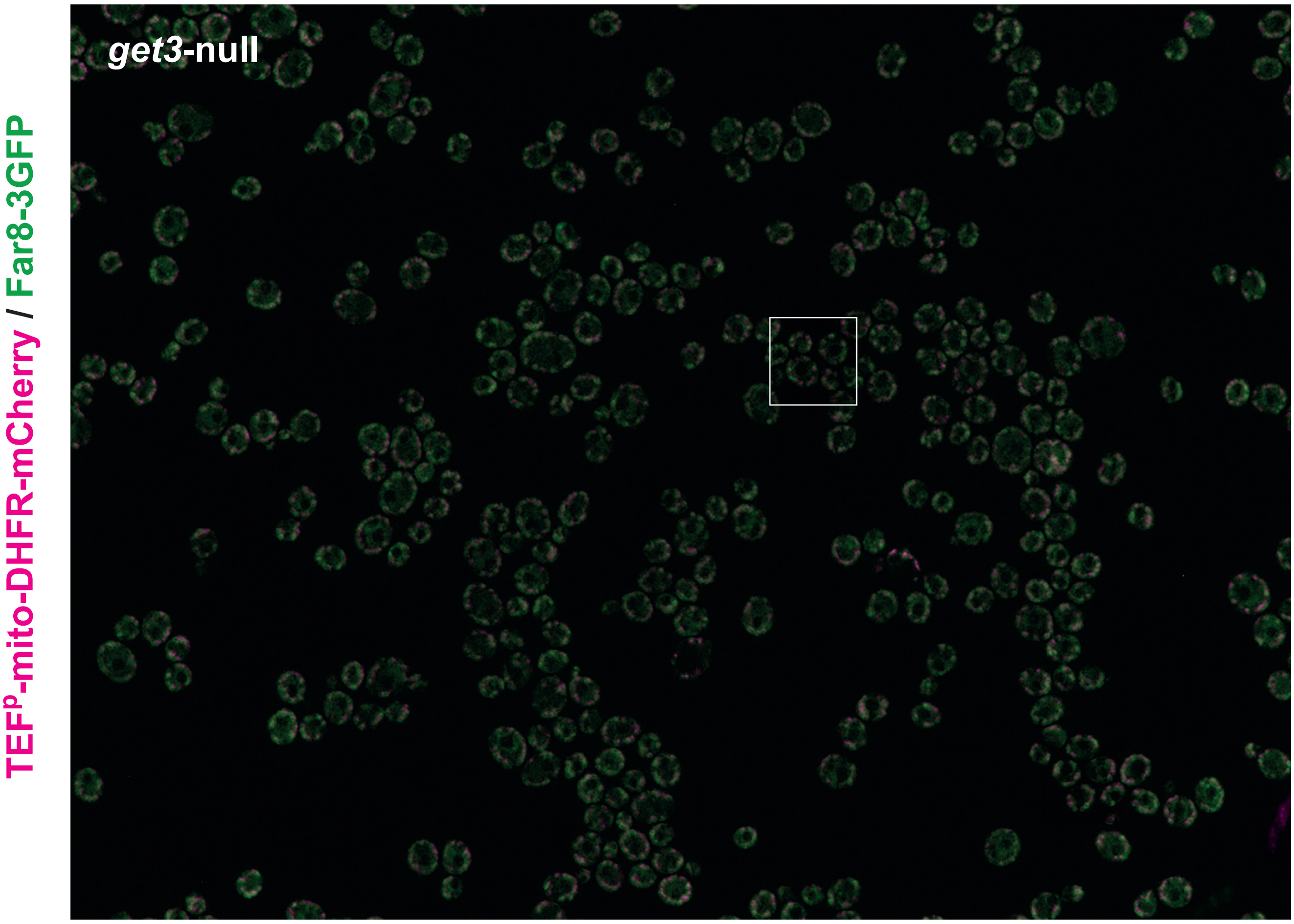

Figure S2B

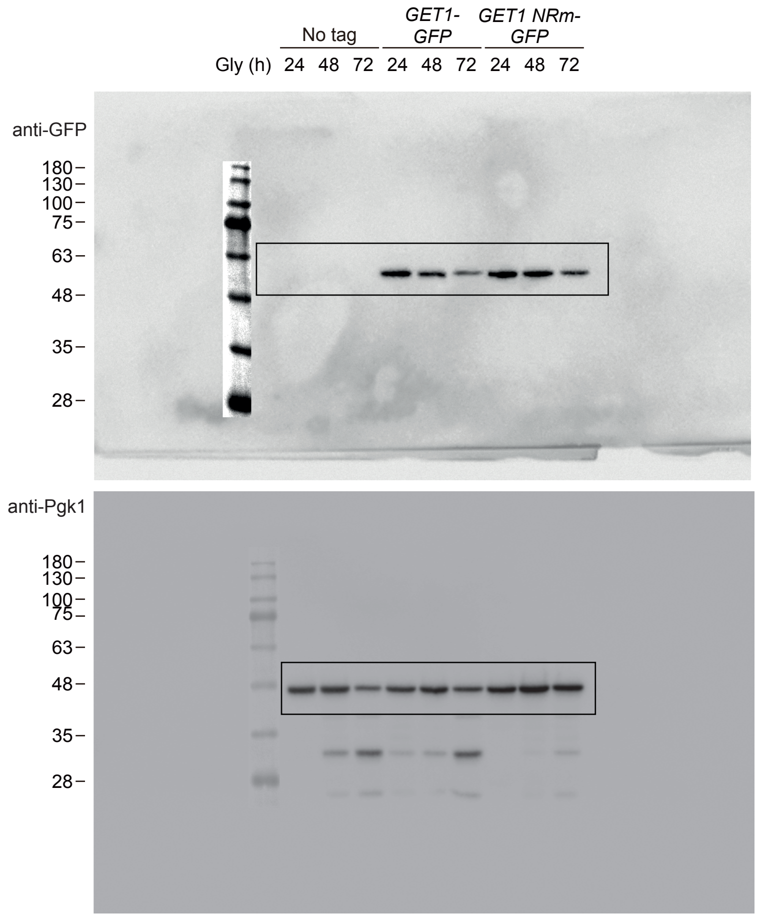

Figure S2C

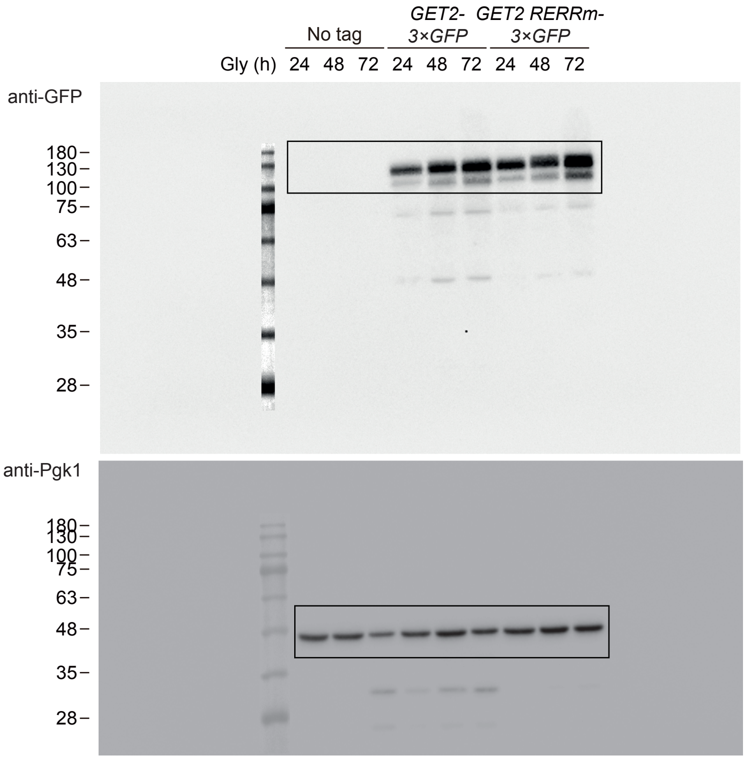

Figure S2D

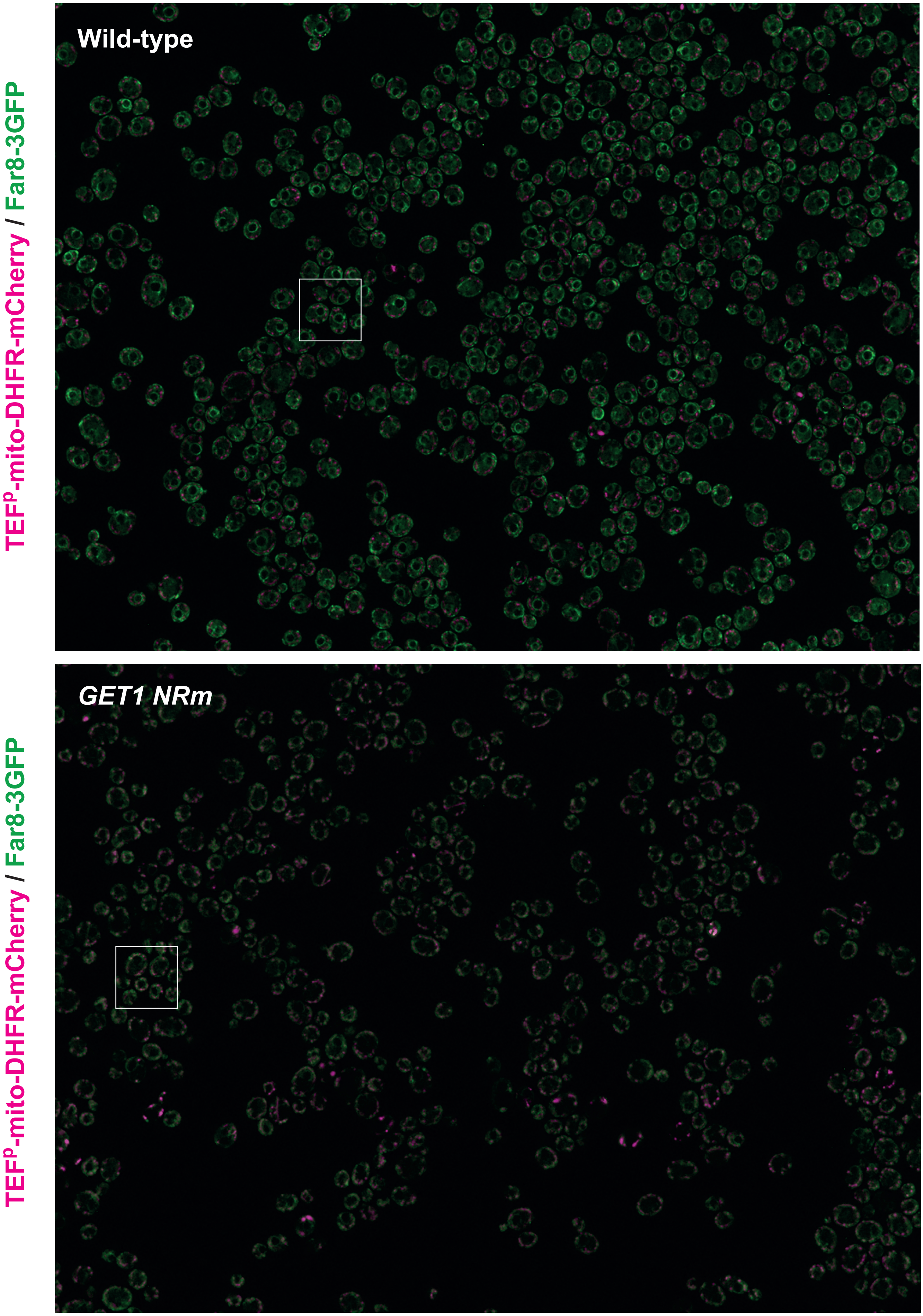

Wild-type
TEFᵖ-mito-DHFR-mCherry / Far8-3GFP
GET1 NRm
TEFᵖ-mito-DHFR-mCherry / Far8-3GFP

Figure S2D

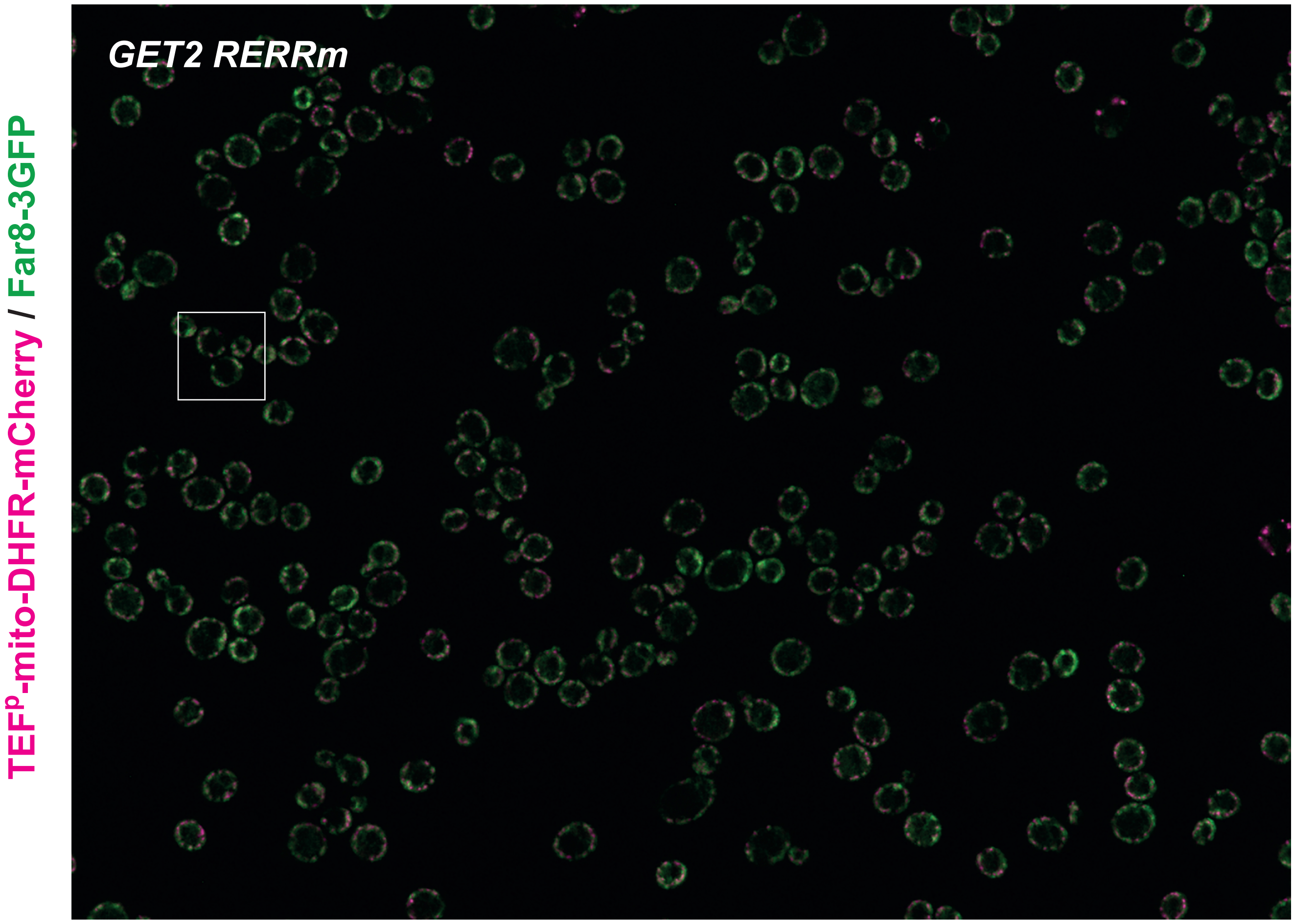

Figure S2E

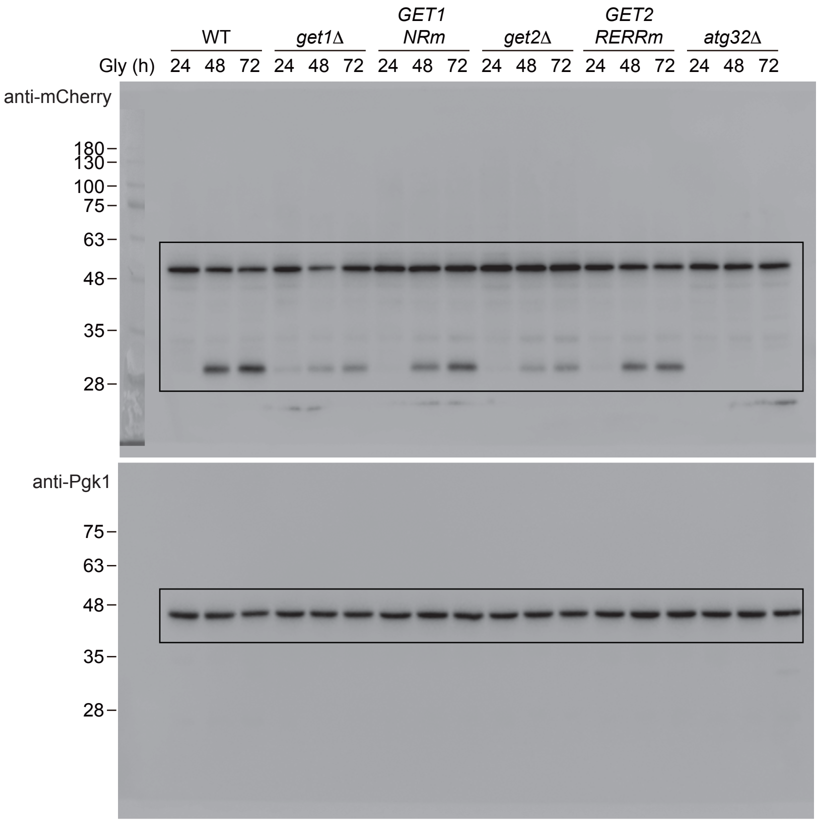
WT
get1Δ
GET1 NRm
get2Δ
GET2 RERRm
atg32Δ
Gly (h) 24 48 72 24 48 72 24 48 72 24 48 72 24 48 72 24 48 72
anti-mCherry
180
130
100
75
63
48
35
28
anti-Pgk1
75
63
48
35
28
