# Supplementary material for: The GET pathway serves to activate Atg32-mediated mitophagy by ER targeting of the Ppg1-Far complex
Source: Life Sci Alliance. 2023 Jan 25;6(4):e202201640. doi: 10.26508/lsa.202201640 (PMC9880027; doi:10.26508/lsa.202201640)
Supplement: Supplementary file 11 [file LSA-2022-01640_SdataFS3.1.pdf]

Onishi et al. Source data related to Figure S3

Figure S3A

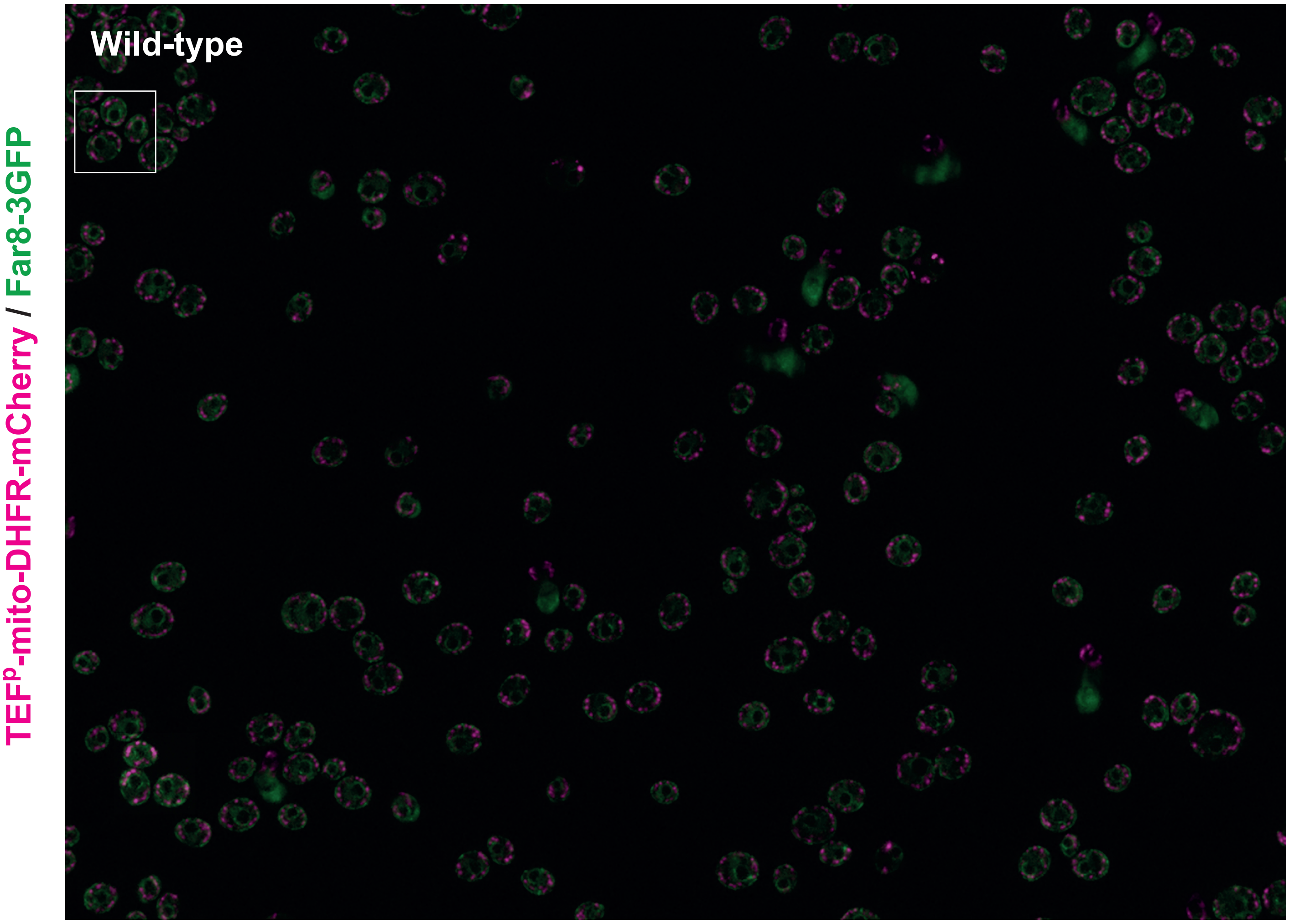

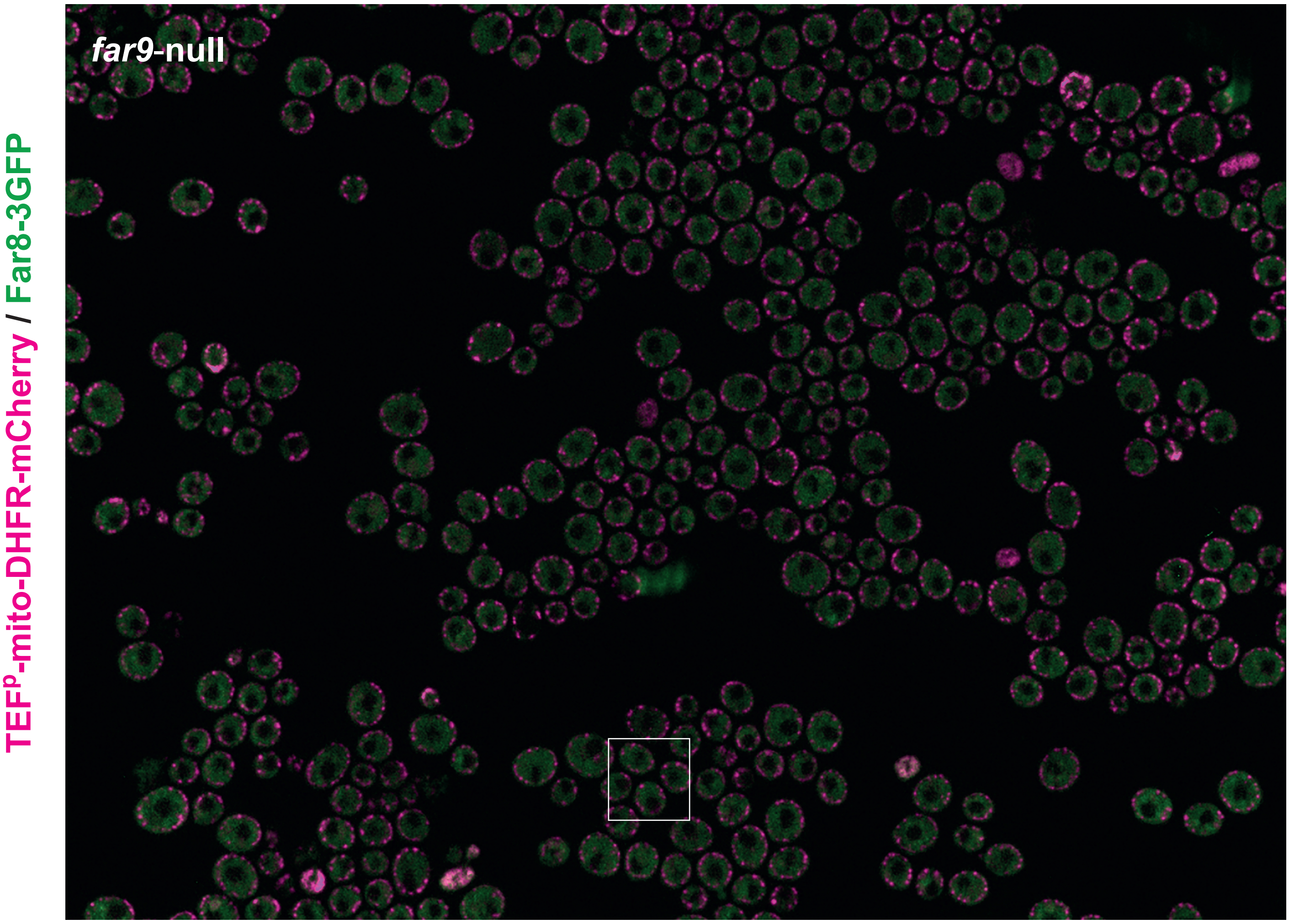

Figure S3A

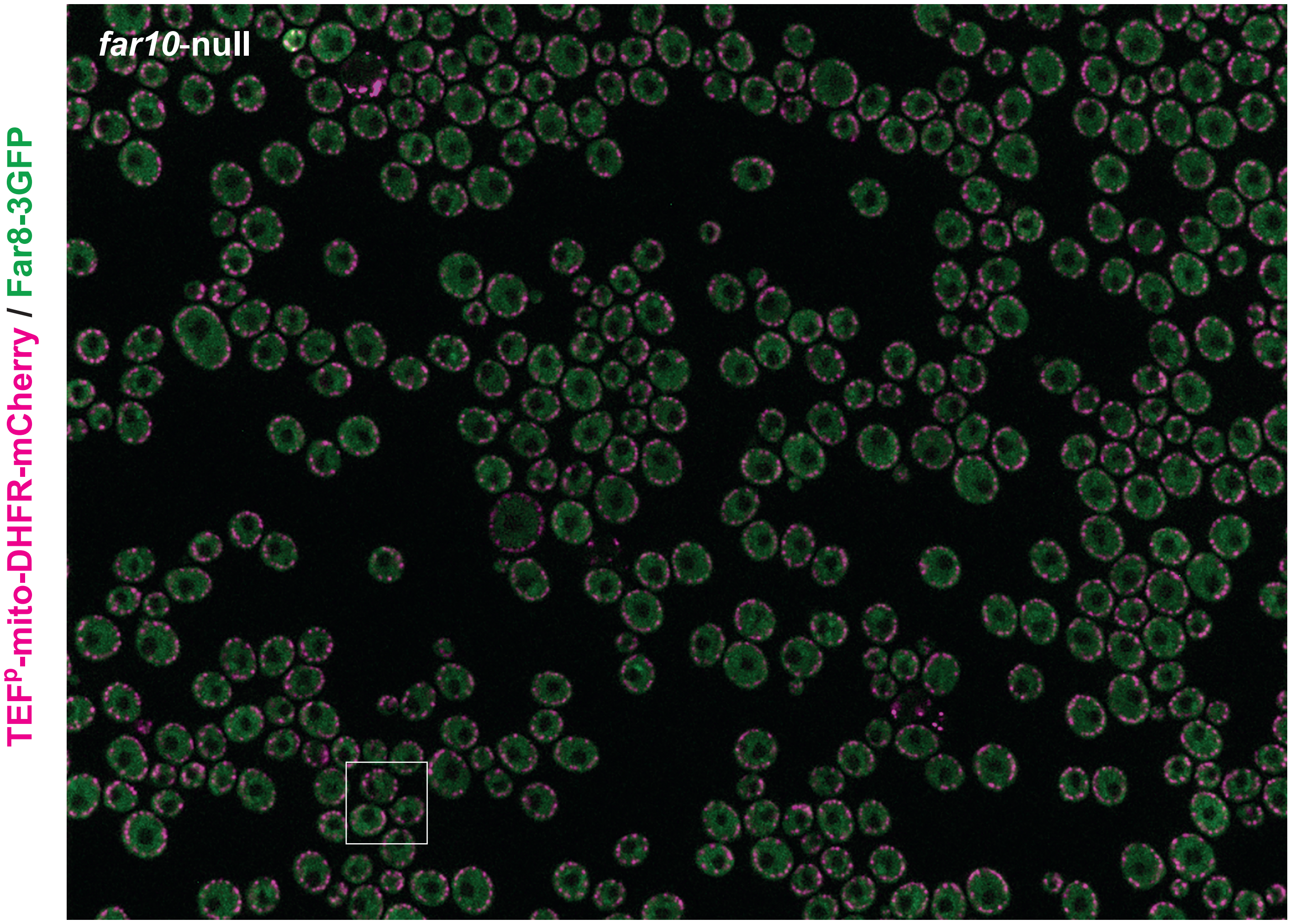
far10-null
TEF^{p}-mito-DHFR-mCherry / Far8-3GFP

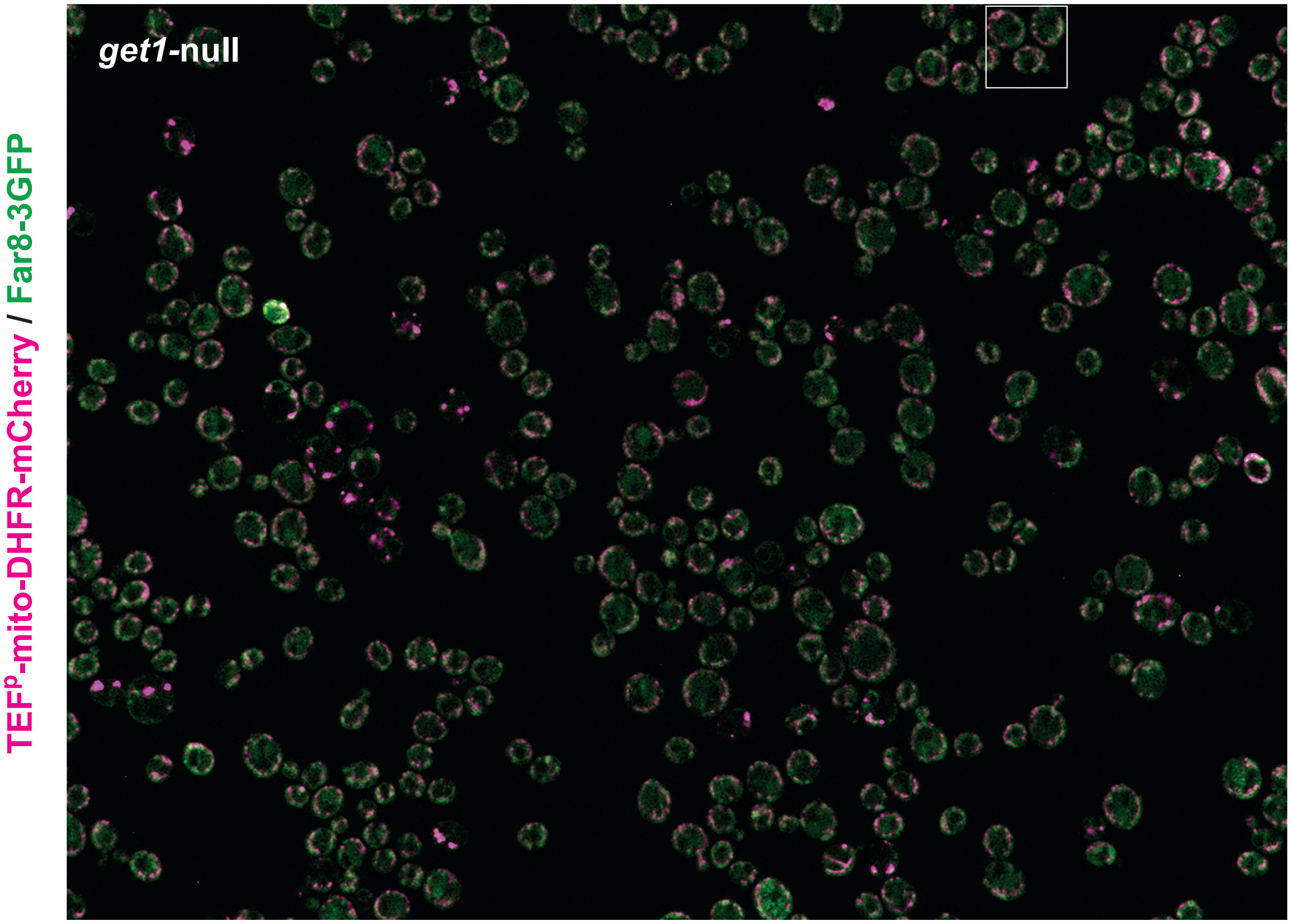
get1-null
TEF^{p}-mito-DHFR-mCherry / Far8-3GFP

Figure S3A

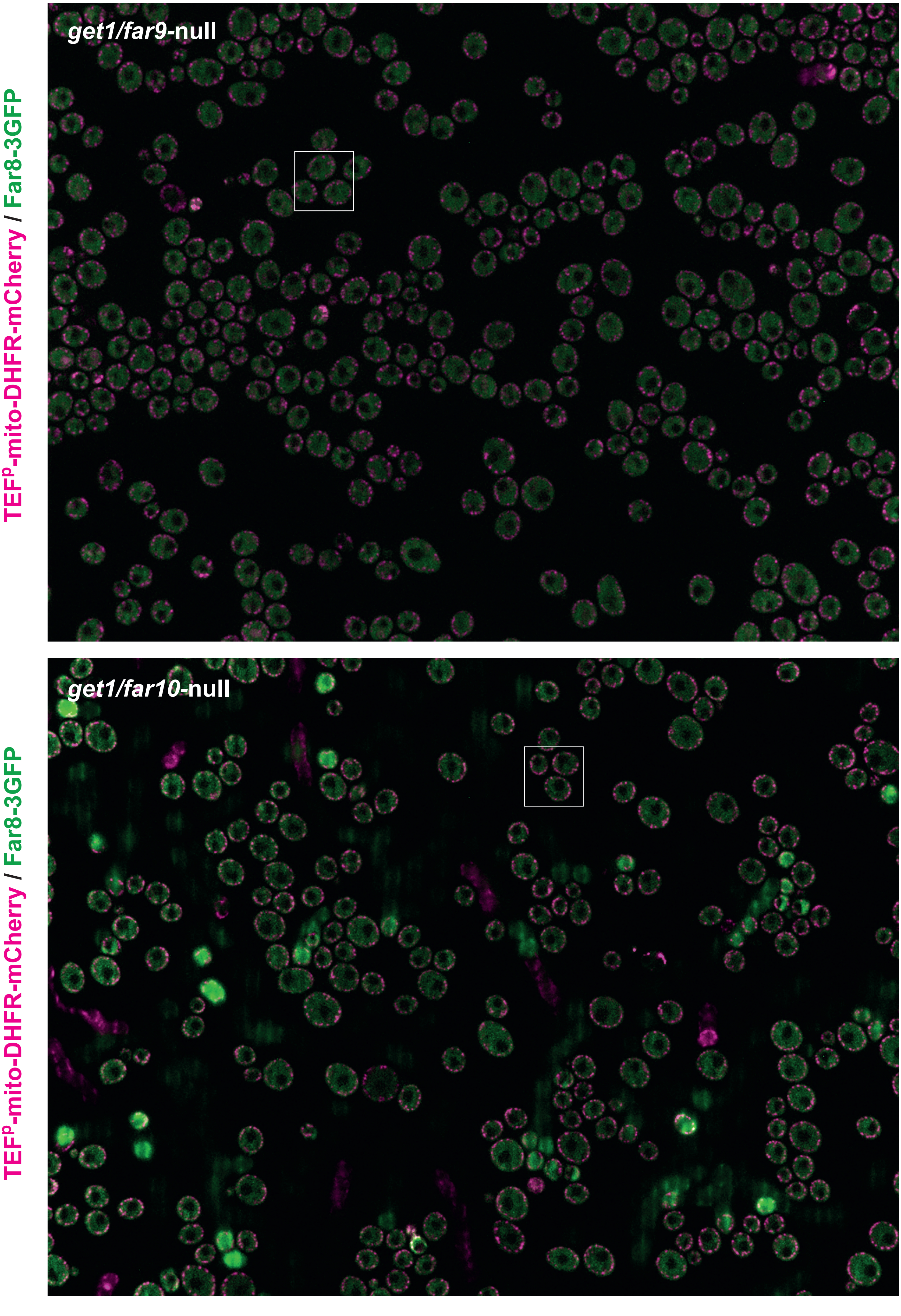
get1/far9-null
TEF^p-mito-DHFR-mCherry / Far8-3GFP
get1/far10-null
TEF^p-mito-DHFR-mCherry / Far8-3GFP

Figure S3C

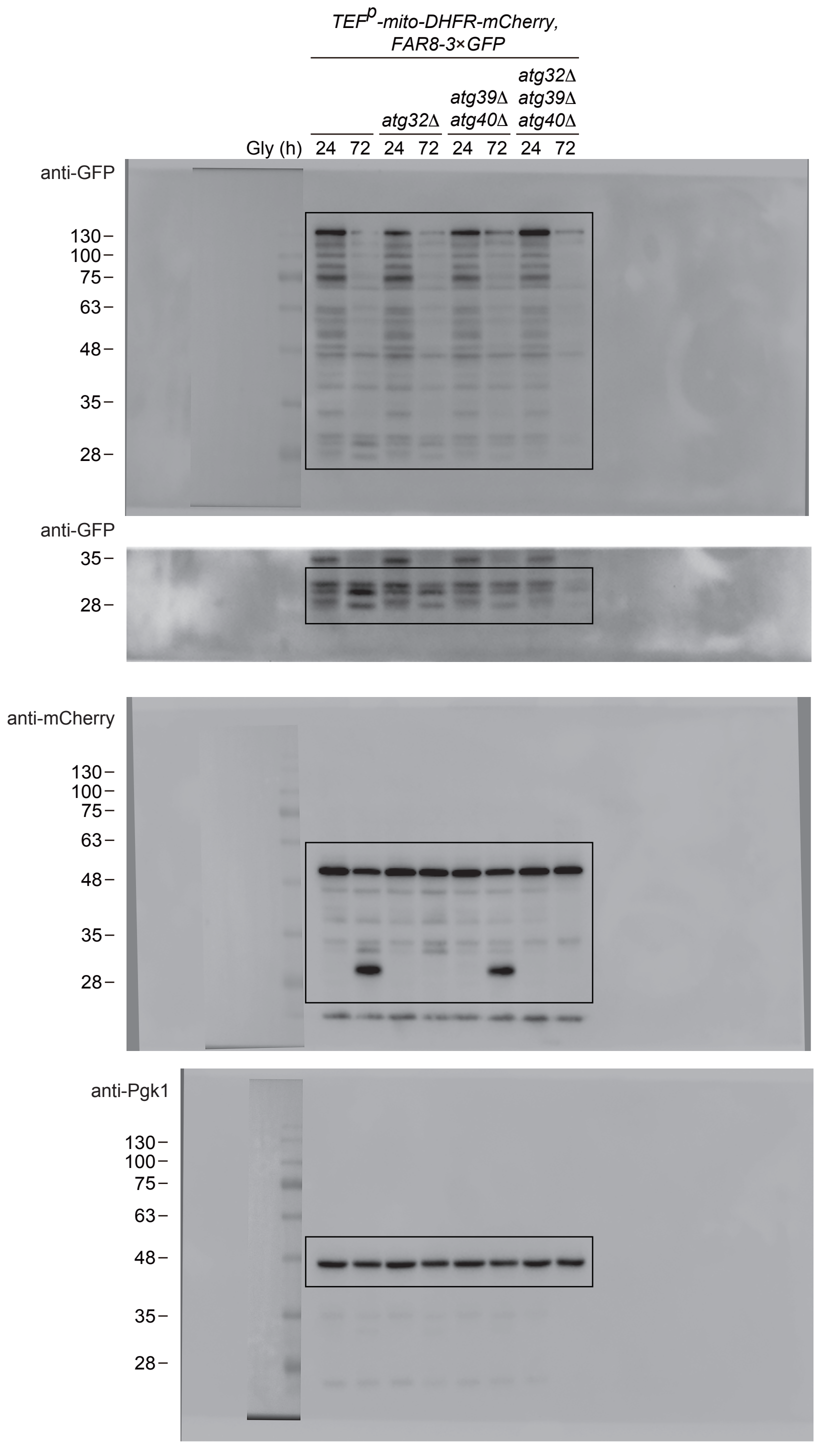

TEP^{p}-mito-DHFR-mCherry,
FAR8-3×GFP
atg32Δ
atg39Δ
atg40Δ
atg32Δ
atg39Δ
atg40Δ
Gly (h) 24 72 24 72 24 72 24 72
anti-GFP
130
100
75
63
48
35
28
anti-GFP
35
28
anti-mCherry
130
100
75
63
48
35
28
anti-Pgk1
130
100
75
63
48
35
28
