# Supplementary material for: The GET pathway serves to activate Atg32-mediated mitophagy by ER targeting of the Ppg1-Far complex
Source: Life Sci Alliance. 2023 Jan 25;6(4):e202201640. doi: 10.26508/lsa.202201640 (PMC9880027; doi:10.26508/lsa.202201640)
Supplement: Supplementary file 17 [file LSA-2022-01640_SdataF5.1.pdf]

Onishi et al. Source data related to Figure 5

Figure 5A

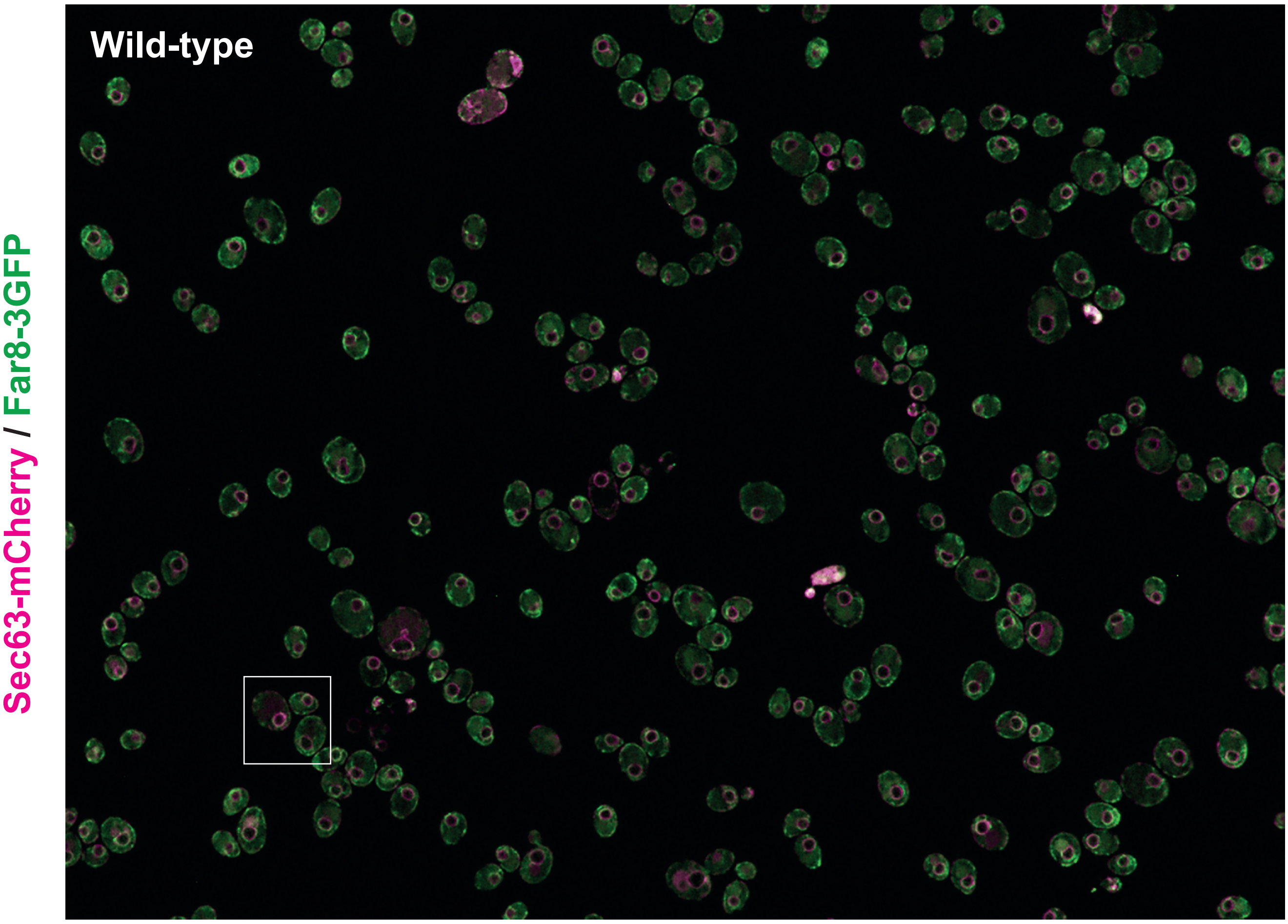

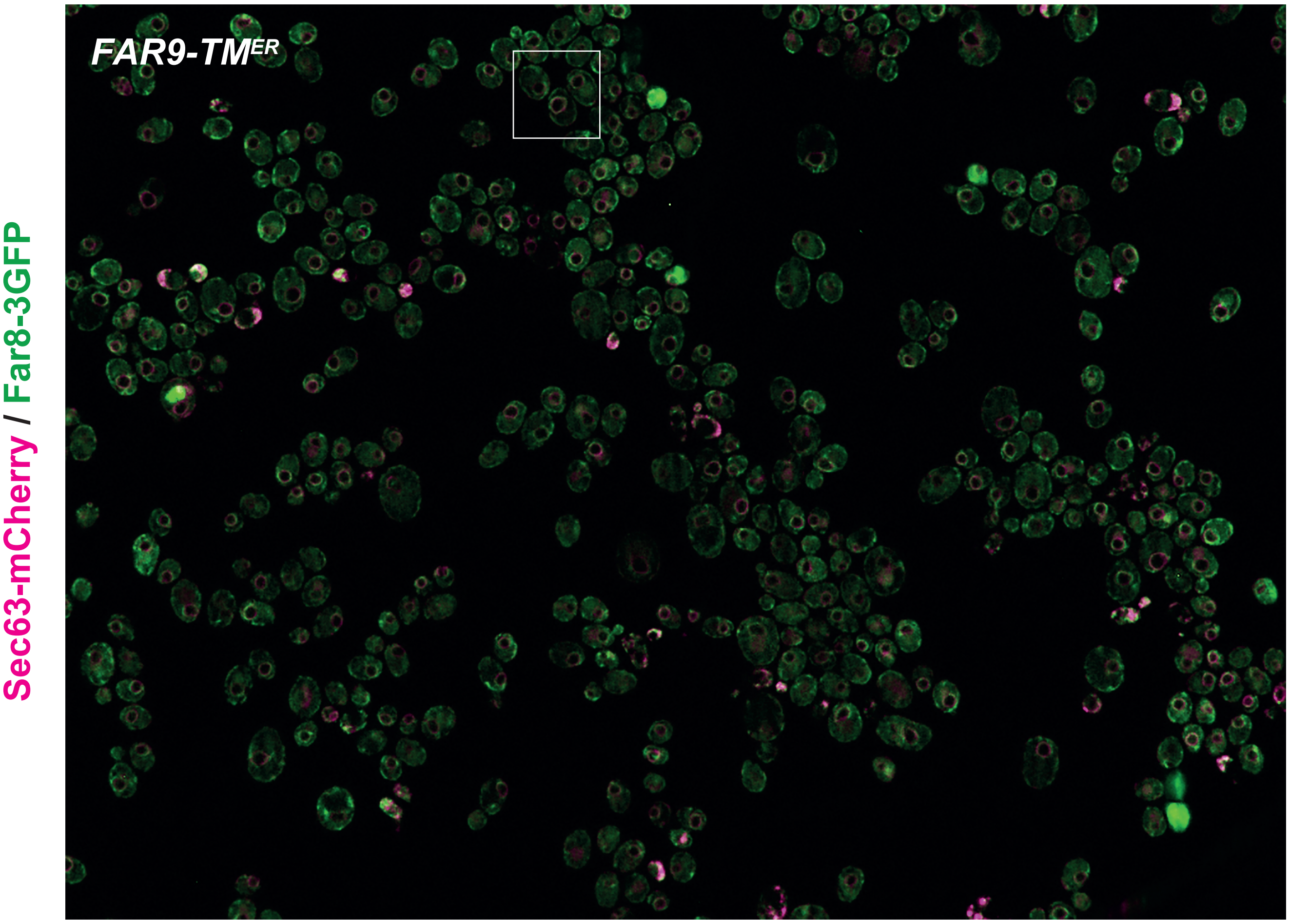

Figure 5A

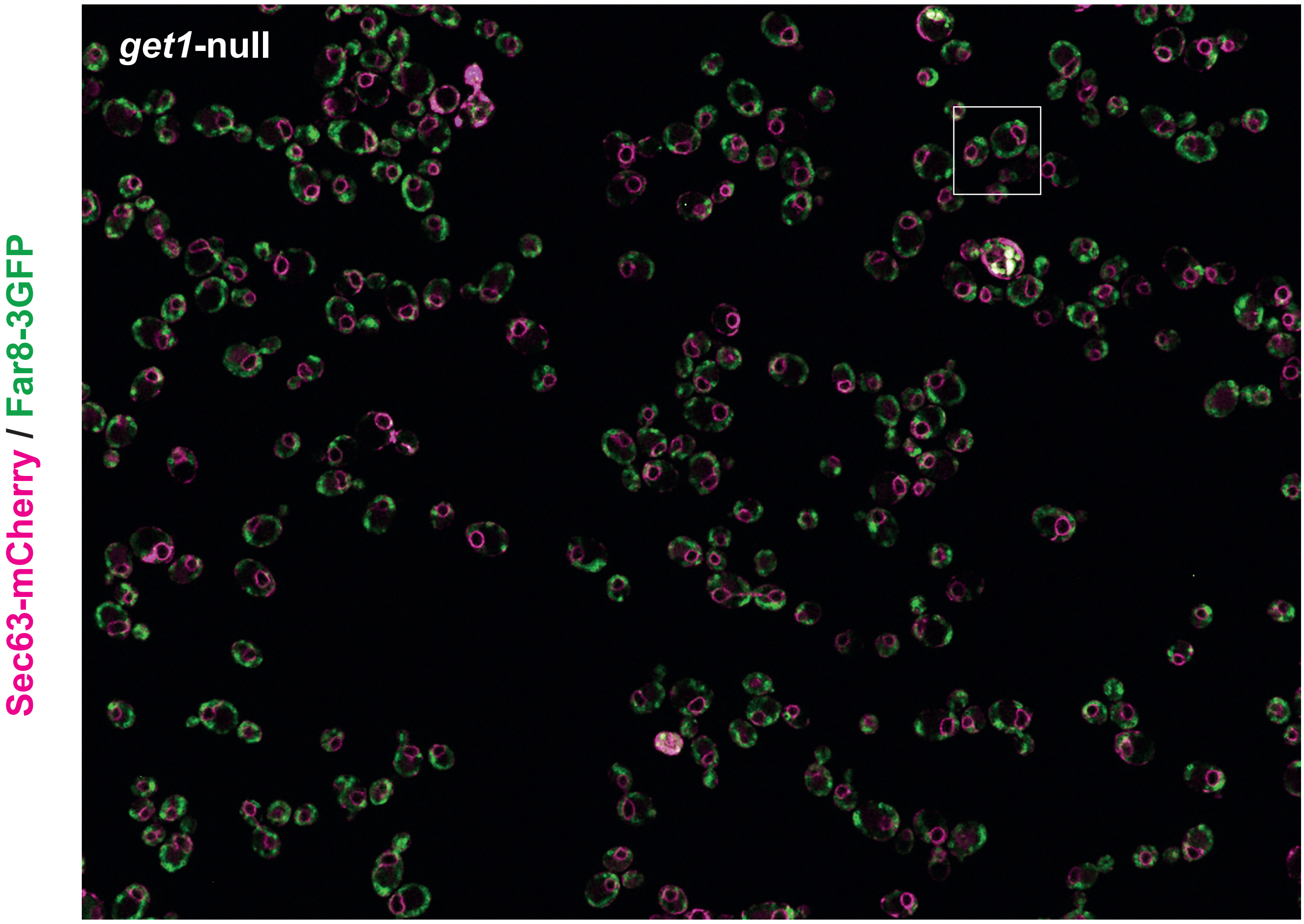
get1-null
Sec63-mCherry / Far8-3GFP

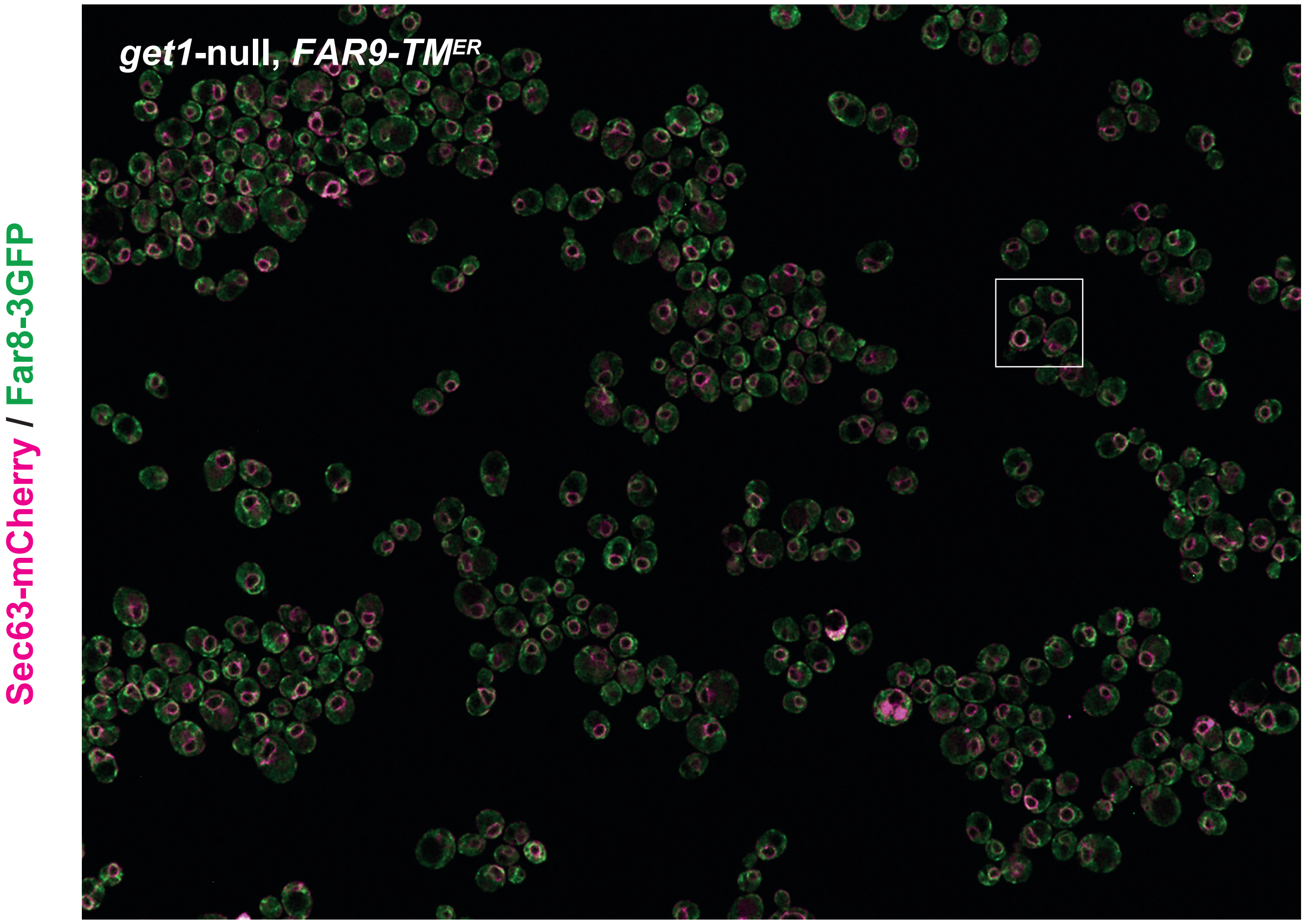
get1-null, FAR9-TMER
Sec63-mCherry / Far8-3GFP

Figure 5A

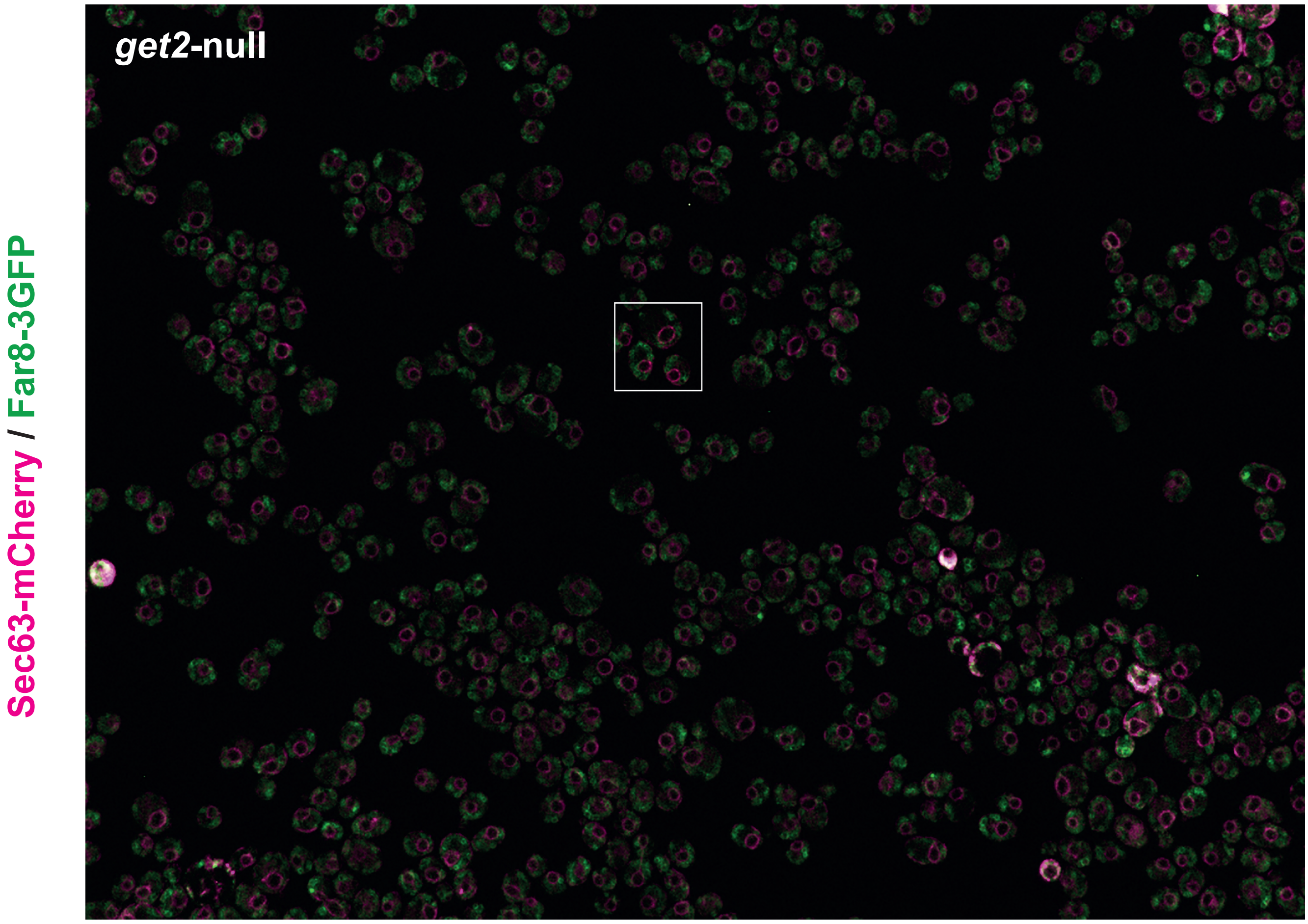
get2-null
Sec63-mCherry / Far8-3GFP

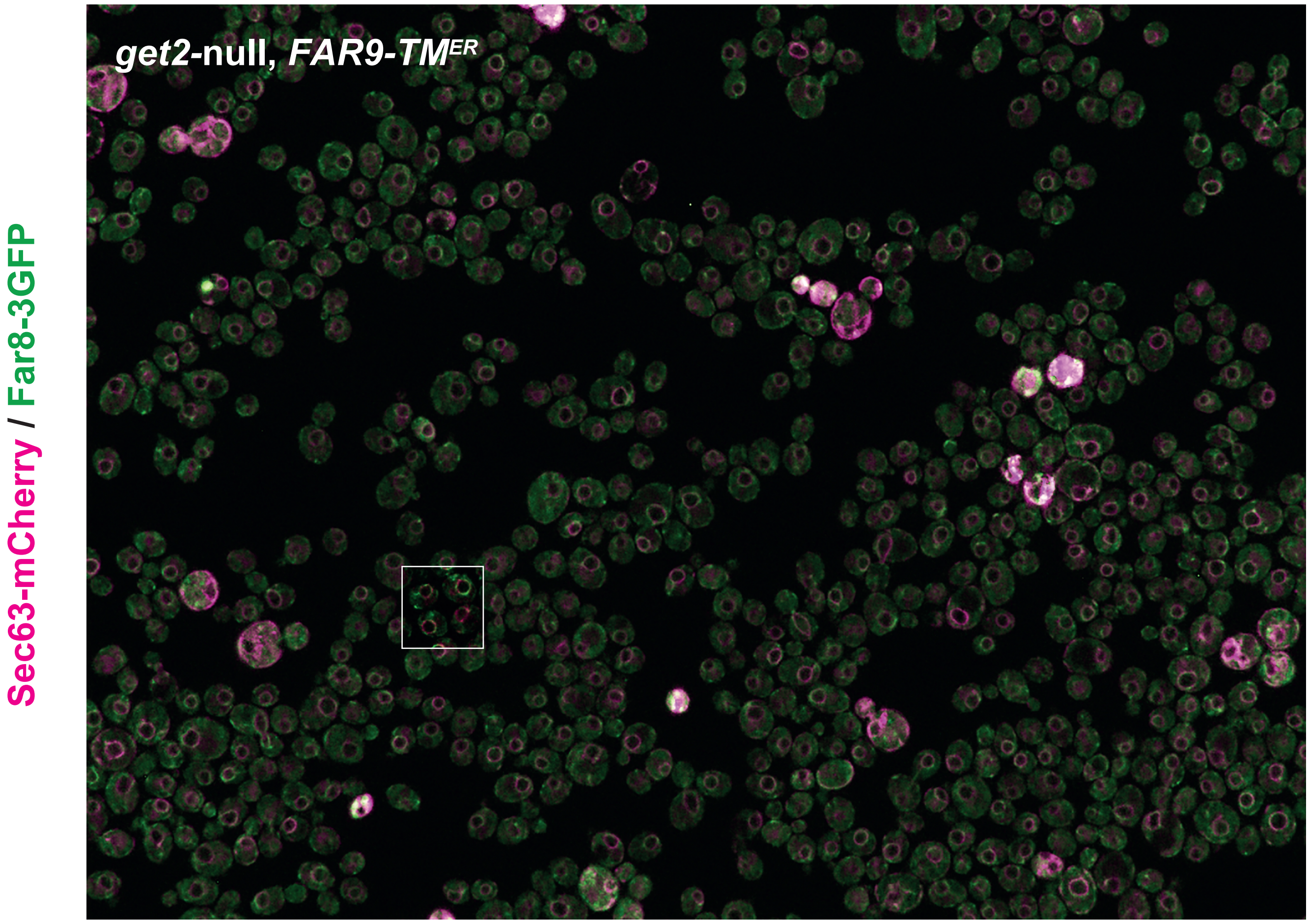
get2-null, FAR9-TMER
Sec63-mCherry / Far8-3GFP

Figure 5D

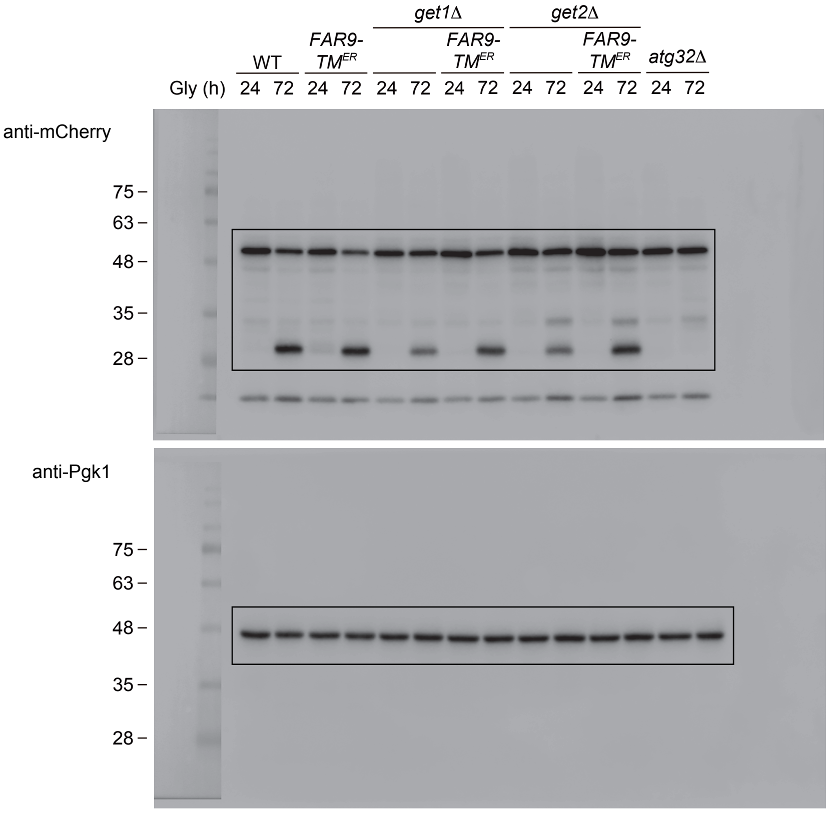

get1Δ
get2Δ
WT
FAR9-TM^ER
FAR9-TM^ER
FAR9-TM^ER
atg32Δ
Gly (h) 24 72 24 72 24 72 24 72 24 72 24 72 24 72
anti-mCherry
75
63
48
35
28
anti-Pgk1
75
63
48
35
28
