# Supplementary material for: The GET pathway serves to activate Atg32-mediated mitophagy by ER targeting of the Ppg1-Far complex
Source: Life Sci Alliance. 2023 Jan 25;6(4):e202201640. doi: 10.26508/lsa.202201640 (PMC9880027; doi:10.26508/lsa.202201640)

Onishi et al. Source data related to Figure 7

Figure 7A

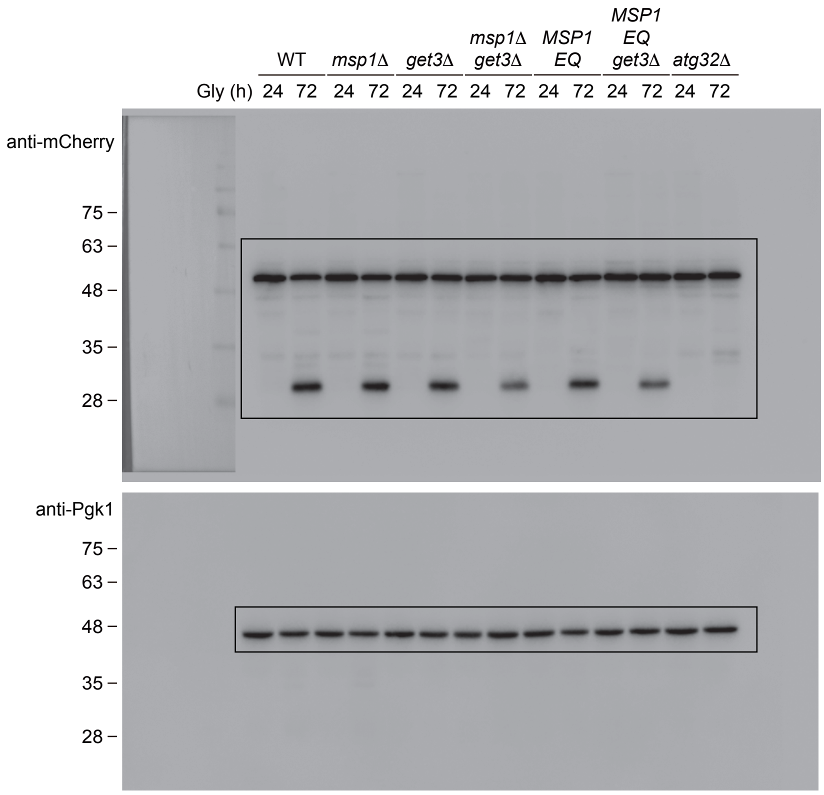

Figure 7C

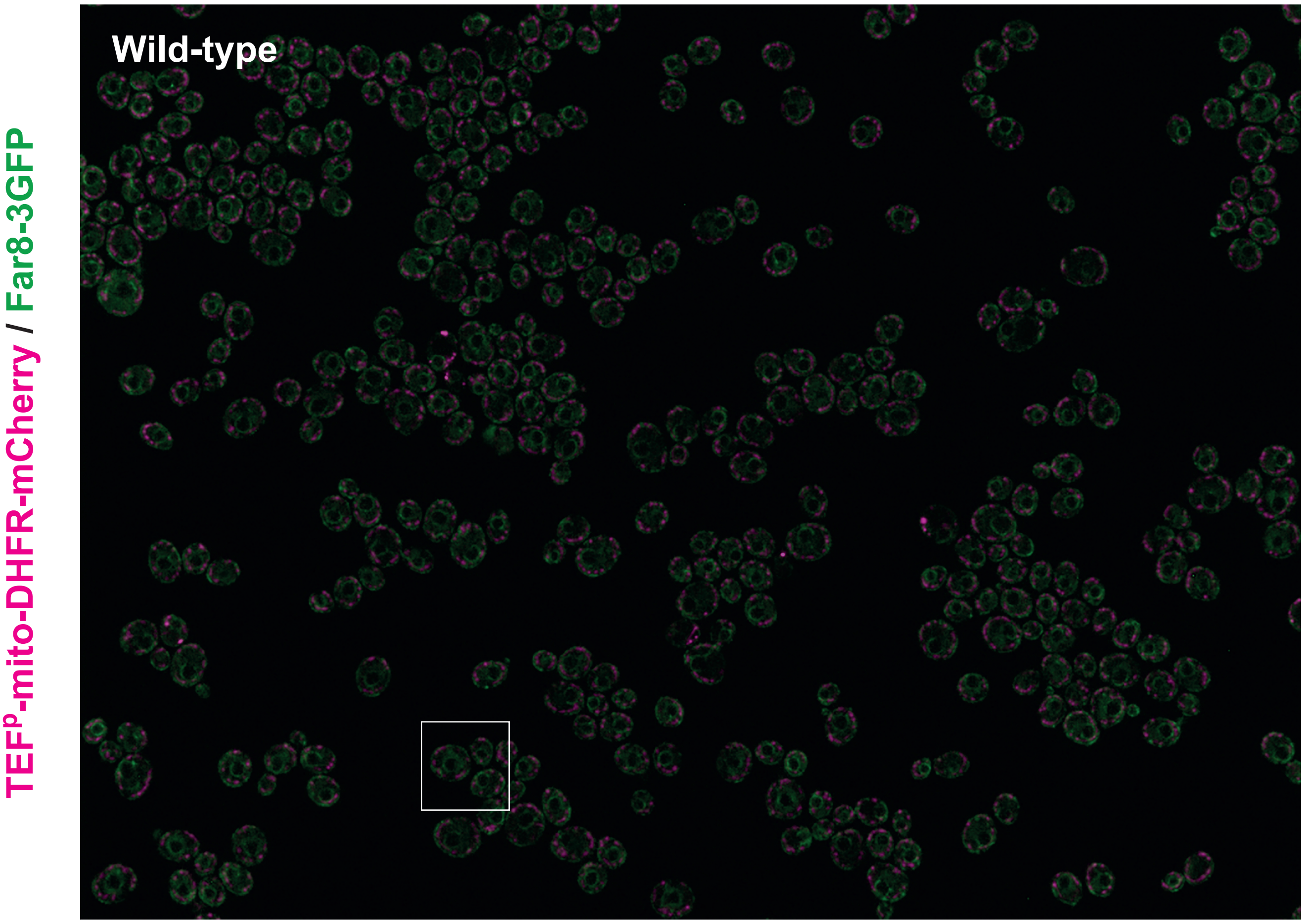
TEF^{p}-mito-DHFR-mCherry / Far8-3GFP
Wild-type

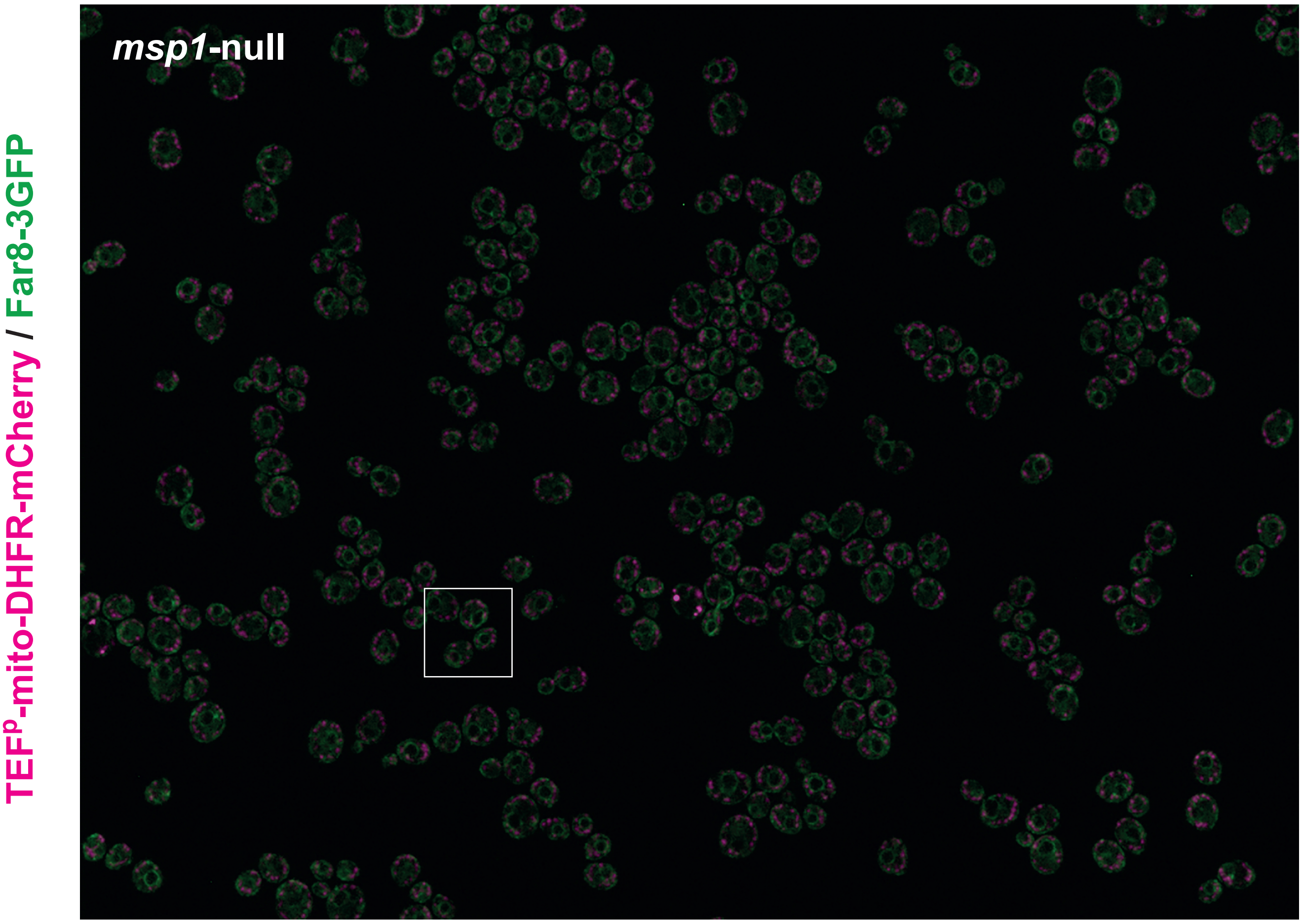
TEF^{p}-mito-DHFR-mCherry / Far8-3GFP
msp1-null

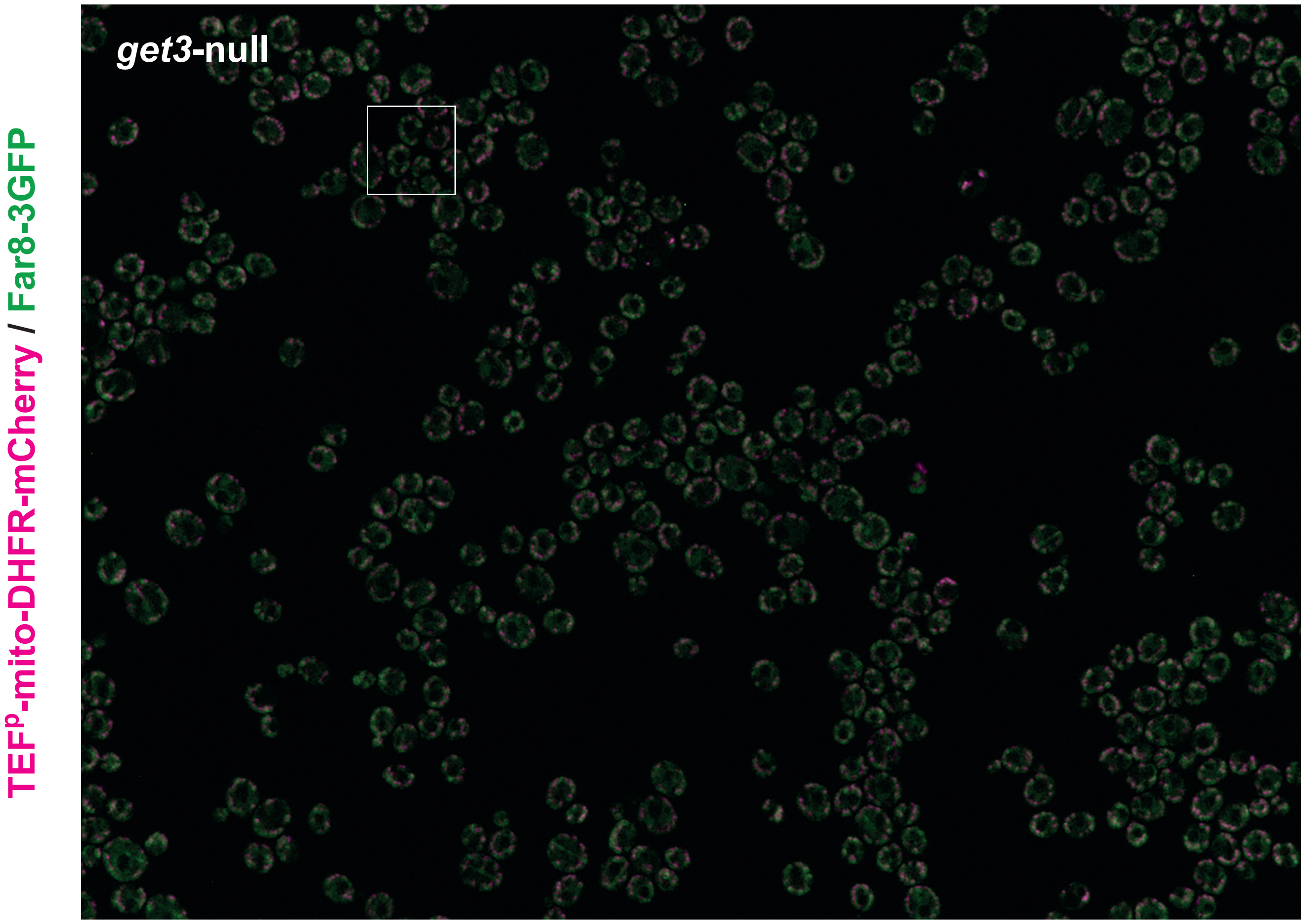
TEFp-mito-DHFR-mCherry / Far8-3GFP
get3-null

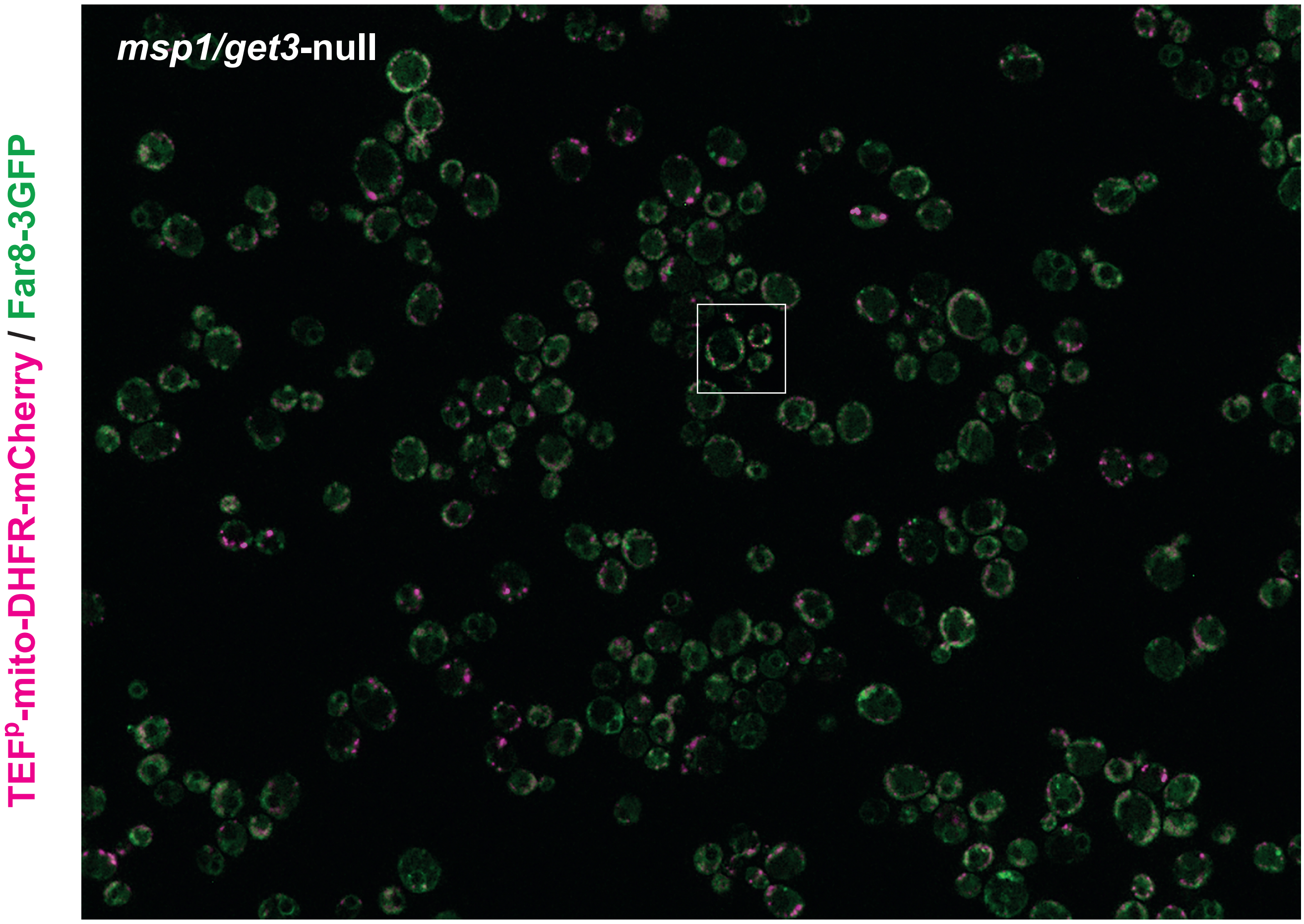
TEFp-mito-DHFR-mCherry / Far8-3GFP
msp1/get3-null

Figure 7E

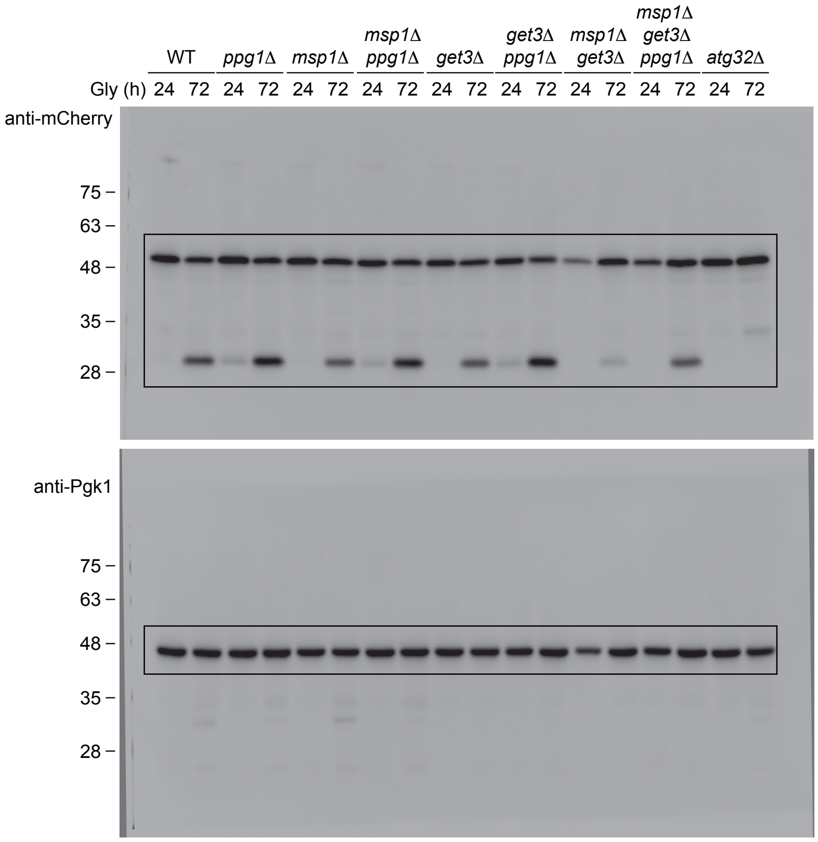

Figure 7G

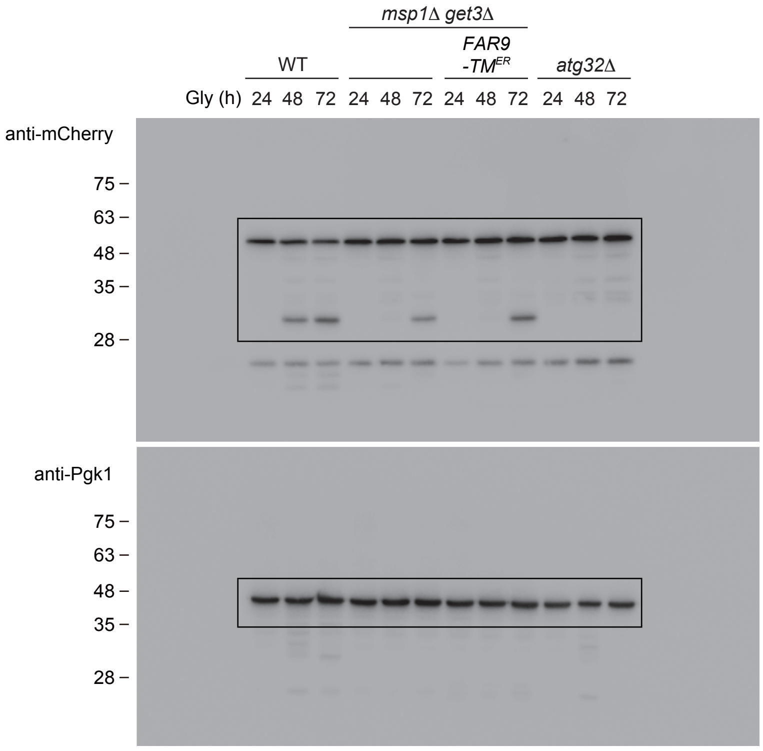

Supplement: Supplementary file 25 [file LSA-2022-01640_SdataF7.1.pdf]
